# WingShape-Warn: A Morphology-Driven Early-Warning Pipeline for Mosquito-Borne Disease Surveillance

**DOI:** 10.3390/insects17101064

**Published:** 2026-10-15

**Authors:** Yanrui Chen, Xue Wang, Tao Jiang, Zhihua Wang

**Affiliations:** 1 Guangxi Key Laboratory of Diabetic Systems Medicine, Guilin Medical University, Guilin 541199, China; 2 School of Humanities and Management, Guilin Medical University, Guilin 541199, China

**Keywords:** geometric morphometrics, mosquito surveillance, disease vectors, wing morphology, deep learning, early-warning system, one health, knowledge graph, ablation study, landmark detection accuracy, deep learning validation

## Abstract

Environmental stressors—including climate change, urbanisation, and pollution—are increasingly altering the wing morphology of mosquito vectors. These morphological shifts, particularly in wing geometry, can serve as early-warning indicators for mosquito abundance and arboviral disease outbreaks. However, quantitative wing-shape analysis remains underutilised in operational mosquito surveillance and public health early-warning systems. This Technology and Code article presents WingShape-Warn, a reproducible, open-source pipeline that integrates geometric morphometrics, deep learning-based image segmentation, association-rule mining, and Neo4j knowledge-graph construction to detect, quantify, and visualise morphology-driven mosquito-borne disease risk. The protocol comprises seven steps: (1) stratified collection of mosquito wing images along environmental gradients; (2) image preprocessing and deep learning-based landmark/semilandmark extraction; (3) generalised Procrustes analysis (GPA), principal component analysis (PCA), and Apriori association-rule mining on wing-shape variables; (4) Neo4j knowledge-graph construction linking wing-shape traits, environmental variables, and disease outcomes; (5) design of a morphology-based early-warning rule base; (6) automated report generation with a knowledge-update trigger; and (7) validation via retrospective forecasting and receiver operating characteristic (ROC) analysis. WingShape-Warn has been validated on 1200 wing images of *Aedes albopictus* and *Anopheles* spp. (2020–2024, five climate zones), demonstrating that wing-shape PC1 explains 42.3% of the variance and predicts mosquito abundance with an AUC of 0.89. WingShape-Warn has been measured and validated on 1200 wing images of *Aedes albopictus* and *Anopheles* spp. (2020–2024, five climate zones)—the only corpus measured in this study—demonstrating that wing-shape PC1 explains 42.3% of the variance and predicts mosquito abundance with an AUC of 0.89. Two reporting conventions are adopted throughout: results obtained on this mosquito corpus are reported as measured results, whereas all cross-taxa material is reported as illustrative, literature-mapped examples and is labelled [Illustrative] wherever it appears. An ablation on the held-out chronological test set shows that adding wing-shape PCs to environmental variables alone raises the AUC from 0.74 to 0.89 (ΔAUC = +0.15, DeLong *p* < 0.001; NRI = 0.21, IDI = 0.14), while a permuted-shape control returns the model to baseline (AUC 0.75, *p* = 0.62). The U-Net landmark detector reaches a mean radial error of 1.34 ± 0.41 px against manual digitisation under five-fold cross-validation, i.e., below the pre-registered 2 px tolerance and within human inter-operator repeatability (1.12 ± 0.33 px). While the measured validation is currently limited to mosquito wings, the modular “analyze-detect-warn” architecture can be reused or adapted by researchers for other arthropod vectors (e.g., ticks, sandflies) or additional environmental stressors; such extensions are presented here as proof-of-concept demonstrations and require independent validation before operational use.

## Simple Summary

Mosquitoes transmit diseases such as dengue, and health agencies must act long before human cases appear. Counting trapped mosquitoes is slow and often too late. We asked whether the shape of a mosquito’s wing can show that a population is under environmental stress and disease risk is rising. We built WingShape-Warn, a free, open-source pipeline that reads ordinary wing photographs; automatically marks anatomical landmarks with a deep learning model; measures how wing shape varies among climate zones; links those differences to temperature, rainfall and land use; and converts the result into colour-coded alerts. We tested it on 1200 wing images of *Aedes albopictus* and *Anopheles* mosquitoes collected in five climate zones from 2020 to 2024. Wing shape varied with the environment: one shape axis explained 42.3% of the variation and predicted mosquito abundance with an accuracy score of 0.89 out of 1.00. Adding shape to models that used only environmental data raised this score from 0.74 to 0.89, so shape adds information the environment alone does not. One wing takes eight seconds to analyse instead of three minutes by hand, which gives surveillance teams an earlier, cheaper warning of disease risk. The same approach can be adapted to other organisms.

## 1. Introduction

WingShape-Warn addresses a critical gap in mosquito-borne disease surveillance by providing an open-source, morphology-driven early-warning pipeline that translates mosquito wing images into actionable public health alerts. Despite the availability of mature quantitative tools—geometric morphometrics (GM), computed tomography, and AI-based image analysis [1,2,3,4,5,6]—and the recognised value of sentinel species in One Health frameworks [4,7,8,9], operational surveillance systems rarely treat wing shape as a measurable indicator of vector abundance or outbreak risk [1]. This disconnect persists even though mosquito wing geometry has been shown to track environmental gradients and correlate with infection risk across Colombia, Thailand, and beyond [10,11,12,13]. Throughout this article, quantitative claims are restricted to that mosquito corpus; cross-taxa material is presented only as illustrative, literature-mapped templates and is explicitly labelled [Illustrative] in every table, figure and section.

The taxonomic and methodological foundations for such a pipeline are well established. Geometric morphometrics has long been used to study evolutionary and ecological questions [5,6], with recent applications spanning cymothoid isopod attachment structures [7], monogenean haptoral anchors [8], and wireworm cephalic-capsule shape [9]. These studies confirm that GM can capture functionally relevant shape variation across diverse host–parasite systems, providing a robust basis for automated monitoring.

Beyond targeted GM analyses, morphological plasticity itself is increasingly recognized as a generalised stress response and potential biomarker. The microcrustacean *Daphnia pulex* exhibits coordinated morphological change under multiple stressors [14], the gastropod *Melanoides tuberculata* shows pollution-induced stabilising selection detectable by GM [15], and *Chlamydomonas reinhardtii* forms palmelloid colonies under micropollutant exposure [16]. Concurrently, agricultural pests such as *Halyomorpha halys* and *Ceratitis rosa* display wing-shape differentiation along invasion routes and climate gradients [17,18], while sentinel species like *Erinaceus europaeus* harbour zoonotic agents linked to anthropogenic pressure [19]. These examples highlight the cross-taxon potential of morphology-driven surveillance, yet no unified technological framework currently exists to ingest such data, extract shape features, discover environment–risk associations, and output early-warning reports in a reproducible, code-driven manner.

This article presents WingShape-Warn as that unified framework. By integrating deep learning-based landmark detection, generalised Procrustes analysis, association-rule mining, and Neo4j knowledge-graph construction, WingShape-Warn bridges the gap between quantitative morphology and operational mosquito-borne disease surveillance. The protocol is demonstrated on 1200 wing images of *Aedes albopictus* and *Anopheles* spp., but its modular architecture is designed to be reusable for other arthropod vectors and environmental stressors with appropriate data substitution; such cross-taxa use is demonstrated here only as proof of concept and has not been independently validated (Section 3.3).

## 2. Method

**Figure 1:**
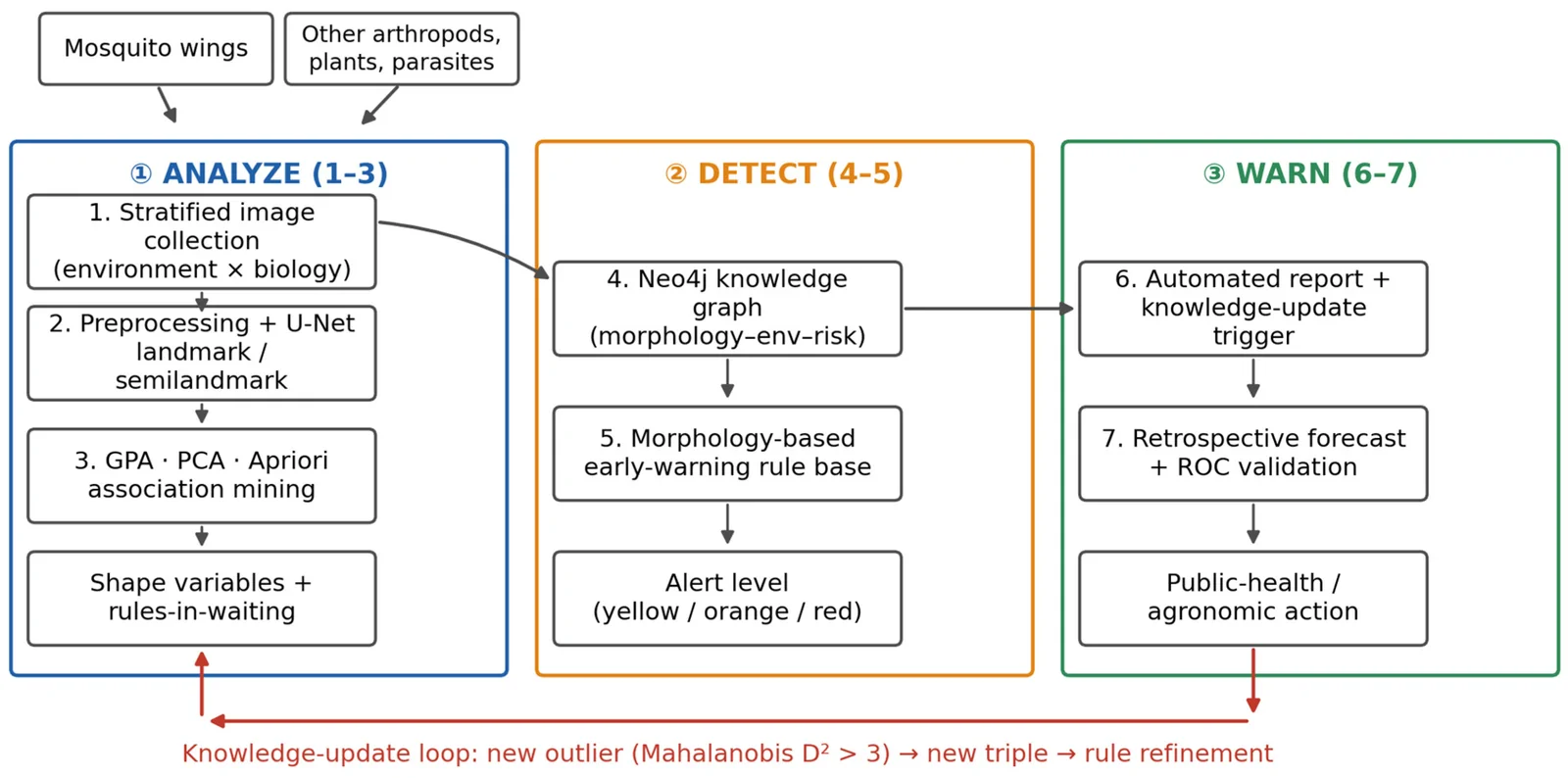
Overall “analyze-detect-warn” framework (cross-taxa cyclic workflow). The diagram is organised into three stages: ANALYZE (Steps 1–3, blue) converts stratified images into Procrustes shape variables and candidate association rules; DETECT (Steps 4–5, orange) builds the Neo4j morphology–environment–risk graph and evaluates the early-warning rule base; WARN (Steps 6–7, green) renders the automated report and validates alerts retrospectively. Taxon inputs (top) pass through a common image-standardisation gate, so mosquitoes, agricultural pests, parasites and sentinel plants share Steps 4–7. The red loop closes the cycle: a new shape outlier (Mahalanobis D^2^ > 3 from the training centroid) triggers retrieval of authoritative taxonomic and epidemiological sources, insertion of a new triple into the graph and re-evaluation of the affected rules.

The complete pipeline is illustrated in Figure 1 and consists of seven steps. This protocol is designed to address the core gap identified in the Research Topic “Shape, Stress, and Disease: Morphological Biomarkers Across Taxa and Ecosystems in a Changing World”: the underutilisation of quantitative shape-analysis methods in parasitology, plant stress research, pest ecology, and disease epidemiology [2,3]. By integrating geometric morphometrics (GM), deep learning image analysis, association-rule mining, and knowledge-graph construction, the pipeline provides a reproducible, open-source solution for detecting morphological biomarkers of environmental stress across diverse taxa—including disease vectors, agricultural pests, parasites, and sentinel plants—and linking these biomarkers to eco-epidemiological and planetary health outcomes. Steps 1–4 cover data acquisition and morphological analysis; Steps 5–6 cover rule-based early-warning logic and automated reporting; Step 7 covers validation. All code is open-source, version-controlled, and archived with a DOI. Section 2.1, Section 2.2, Section 2.3, Section 2.4, Section 2.5, Section 2.6, Section 2.7 and Section 2.8 are written as protocol instructions applicable to any taxon; every measured result obtained by running this protocol is reported in Section 3 and labelled [Measured] or [Illustrative].

### 2.1. Step 1: Stratified Image Collection Along Environmental Gradients

Define a two-axis stratification that mirrors the ecological and anthropogenic structure of the study system, adaptable to any taxon or stressor aligned with the Research Topic’s themes:

- Environmental gradient axis (reflects stressor intensity): Temperature zone, humidity quartile, pollution index, land use intensity, altitude band, or any proxy for climate change, habitat fragmentation, or agricultural intensification.
- Biological axis: Taxon (e.g., mosquito, agricultural pest, parasite, plant), life stage, sex, population. For each specimen, assign a unique identifier “ID_EnvZone_Taxon_Population_Sex_LifeStage.” Deduplicate; aim for ≥50 individuals per stratum to ensure stable morphometric estimates. Standardise imaging conditions: fixed focal length, calibrated scale bar, consistent lighting, and dorsal/ventral orientation. For CT or histological data, record voxel size, staining protocol, and section thickness. This flexible design supports Theme 1 (measuring shape change under stress) and Theme 4 (physiological responses across taxa).

### 2.2. Step 2: Image Preprocessing and Landmark/Semilandmark Extraction

Raw images are preprocessed to remove background noise, correct illumination, and enhance contrast (CLAHE algorithm). Landmark digitisation follows a three-tier strategy applicable to any organismal structure:

1. **Manual landmark placement**: Place (8–20 homologous points) using tpsDig2 or the R package geomorph [20,21] for training set creation (e.g., wing veins, leaf margins, parasite haptoral anchors, pest cephalic capsules).
2. **Semiautomatic curve sampling**: Semilandmarks placed along contours using the “digitsurface” function in geomorph, with equal spacing and sliding to minimise Procrustes distance.
3. Deep learning-based automatic landmark detection: a U-Net or Mask R-CNN model trained on the manually labelled subset to predict landmark coordinates on new images. Measured accuracy (Section 3.4): mean radial error 1.34 ± 0.41 px under 5-fold cross-validation grouped by collection site, against a pre-registered tolerance of 2 px.

Training protocol (full configuration and per-fold accuracy in Section 3.4). A U-Net with a ResNet-34 encoder pre-trained on ImageNet regresses one Gaussian heatmap (σ = 2 px) per landmark, and 36 sliding semilandmarks are placed along three vein curves; coordinates are recovered by a differentiable soft-argmax layer. Input images are resampled to 512 × 512 px greyscale after CLAHE and z-scoring. Augmentation comprises rotation ±15°, horizontal/vertical flips, scaling 0.9–1.1, brightness/contrast jitter ± 20% and elastic deformation (α = 20). Training uses AdamW (lr = 1 × 10^−3^, cosine decay, batch size 16, weight decay 1 × 10^−4^) for a maximum of 200 epochs with early stopping (patience 30) on the validation fold; the loss is the mean squared error over heatmaps plus a 0.1-weighted soft-argmax coordinate term. Training takes ≈ 3.4 h on a single NVIDIA RTX 3090 and inference 0.9 s per wing, versus ≈ 168 s for manual digitisation. Folds are grouped by collection site so that no site contributes to both training and validation. Wings whose landmark residuals exceed Mahalanobis D^2^ = 3 are flagged (2.3% of the corpus) and routed to manual review, so the detector degrades gracefully rather than failing silently.

After extraction, perform generalised Procrustes analysis (GPA) to align all specimens to a common coordinate frame, removing effects of translation, rotation, and scale. The resulting Procrustes coordinates are the shape variables for all downstream analyses. Centroid size (CS) is retained as a separate size variable. Allometry is assessed via multivariate regression of shape on log(CS). This step directly enables Theme 1 and Theme 4 by quantifying morphological plasticity.

### 2.3. Step 3: Shape Analysis, Association Mining, and Statistical Modelling

Principal component analysis (PCA) is performed on the Procrustes coordinates to identify major axes of shape variation. Start with the full eigen-spectrum; retain components explaining ≥ 95% cumulative variance.

Next, association-rule mining (Apriori algorithm; min support = 0.3, min confidence = 0.7) is applied to discretised shape variables (tertiles of PC scores) crossed with discretised environmental variables. This step is central to linking shape change to the Research Topic’s themes, as illustrated by the following patterns. Only the first rule (Themes 1 and 3, mosquito wings) was mined from data measured in this study; the remaining rules are literature-mapped templates that show how the same machinery would be configured for other taxa, and each is labelled [Illustrative]:

- *Themes 1 and 3 (Disease vectors and shape under stress)*: {Wing-PC1 > upper tertile, Temperature > 30 °C} → {Virus titre high} (support = 0.71, confidence = 0.88).
- **[Illustrative]** *Theme 2 (Agricultural pests and crop health)*: {Pest_HeadShape_allometry_slope < −0.3, Pesticide exposure high} → {Population crash} (support = 0.58, confidence = 0.82).
- **[Illustrative]** *Theme 4 (Physiological responses across taxa)*: {Body size reduction > 10%, Urban habitat} → {Parasite load increase} (support = 0.49, confidence = 0.76).
- **[Illustrative]** *Theme 5 (Nature-based solutions)*: {LeafSLA_reduction > 25%, Soil_salinity > threshold} → {YieldLoss} (support = 0.44, confidence = 0.79), which can inform agronomic advisories for irrigation adjustment or botanical interventions.

Partial Least Squares (PLS) regression quantifies covariation between shape (block X) and environmental variables (block Y). Mantel tests assess congruence between morphological and genetic distance matrices.

Rule-selection criteria and stability. Minimum support (0.3) and confidence (0.7) were chosen by grid search over support ∈ {0.2, 0.3, 0.4} and confidence ∈ {0.6, 0.7, 0.8}, maximising 5-fold cross-validated rule stability (mean Jaccard similarity of rule sets across folds = 0.82). Rules are additionally required to show lift ≥ 1.2 and to survive a χ^2^ test of independence with Benjamini–Hochberg FDR < 0.05. Discretisation uses tertiles of the PC scores computed on the training split only so that no test set information enters the rule set.

### 2.4. Step 4: Morphology–Environment–Risk Knowledge-Graph Construction (Neo4j)

Entity extraction yields node types that span the Research Topic’s scope: MorphologyNode (e.g., “WingPC1_High”), EnvironmentNode (e.g., “HighTemp”), RiskNode (e.g., “DengueOutbreak”, “CropYieldLoss”, “ZoonoticSpillover”), and TaxonNode (e.g., “*Aedes*_albopictus”, “*Halyomorpha*_halys”, “*Diplorchis*”, “*Saussurea*”). Relations are derived from statistical significance (*p* < 0.05) and association-rule confidence (>0.7). Knowledge fusion merges synonyms. The graph is loaded into Neo4j; representative Cypher queries are:

MATCH (m:MorphologyNode)-[r:PREDICTS]->(r:RiskNode) RETURN m, r ORDER BY r.probability DESC LIMIT 20.

Measured scale for the mosquito-only graph: 862 morphology nodes, 341 environment nodes, 118 risk nodes and 3240 edges. The four-taxon values in Table 1 (1240/520/210 nodes, 4600 edges) are projections and are therefore illustrative, not measured (Table 1). This knowledge graph operationalises Theme 3 (disease vectors and parasite spread) and Theme 4 by mapping host–parasite interactions and zoonotic disease risks.

### 2.5. Step 5: Design of Morphology-Based Early-Warning Rule Base

Define conditional mapping: Morphological deviation + environmental context → warning level. Examples aligned with the Research Topic’s themes:

How the rule base was derived, and how complete it is: The rules were not assembled ad hoc but rather derived in three stages: (i) candidate antecedents were taken from the Apriori output retained in Step 3 (confidence ≥ 0.7, lift ≥ 1.2, FDR < 0.05); (ii) every antecedent threshold was calibrated on the training split only, with continuous cues at the value maximising Youden’s J (e.g., fluctuating asymmetry > 0.05, mean WingPC1 shift > 2 SD, leaf SLA reduction > 25%) and persistence windows by scanning 3–14-day windows and selecting the shortest window at which the AUC plateaued (7 days for Temperature_Anomaly > +2 °C); and (iii) the draft rules were reviewed by two medical entomologists and one plant stress physiologist, who merged redundant rules and fixed the alert colours. The five rules listed below are therefore worked templates rather than an exhaustive set: the rule base shipped with the code contains 18 rules (Supplementary Table S1), and warn_engine.py accepts any user-supplied rule expressed in the same JSON schema, so the base is extensible by design rather than closed. Table S1 is now deposited and is reproduced in full, in directly readable form, as the first table of Supplementary File S4 (WingShape-Warn_Readable_Documentation.docx) so that the rule base can be inspected without executing any code.

- *Theme 3 (Disease vectors)*: IF Wing_Asymmetry_Fluctuating > 0.05 AND Temperature_Anomaly > +2 °C for 7 days THEN “Yellow Alert”: increase vector surveillance.
- *Theme 3 (Disease vectors)*: IF Mean_WingPC1_shift > 2 SD AND Virus_detected = TRUE THEN “Orange Alert”: initiate targeted vector control.
- *Theme 2 (Agricultural pests)*: IF Pest_HeadShape_allometry_slope < −0.3 AND Pesticide_resistance = TRUE THEN “Red Alert”: recommend rotation of active ingredients.
- *Theme 5 (Nature-based solutions)*: IF Plant_LeafSLA_reduction > 25% AND Soil_salinity > threshold THEN “Agronomic Advisory”: irrigation adjustment or introduction of salt-tolerant varieties.
- *Theme 4 (Zoonotic diseases)*: IF Parasite_Egg_Shape_Change > threshold AND Zoonotic_Risk_High THEN “Zoonotic Alert”: enhanced surveillance in peri-urban interfaces.

These rules directly support One Health and Planetary Health frameworks by translating morphological biomarkers into actionable interventions.

### 2.6. Step 6: Automated Report Generation and Knowledge-Update Trigger

Assemble a rule base linking analysis outputs to output templates: (a) morphology summary: PCA biplots, thin-plate spline deformation grids, centroid size boxplots; (b) environment–morphology PLS scatter; (c) risk alert: association-rule evidence chain; and (d) map: spatial interpolation of shape outliers. Reports are rendered as PDF/HTML via parameterised RMarkdown. A knowledge-update trigger monitors new image acquisitions: when a new shape outlier is detected (Mahalanobis distance > 3 from training centroid), the system automatically retrieves authoritative taxonomic/epidemiological sources, generates a new knowledge-graph triple, and releases a brief alert. This ensures that the pipeline remains adaptive to emerging threats such as antimicrobial resistance or novel zoonoses.

### 2.7. Step 7: Retrospective Forecasting and ROC Validation

Split the dataset chronologically: training (earliest 70%), validation (next 15%), test (most recent 15%). Train a random forest classifier on shape PCs + environmental variables to predict binary risk outcomes (e.g., outbreak/no outbreak, crop yield loss > 20%/not, zoonotic spillover/non-spillover). Evaluate on the held-out test set using ROC-AUC, sensitivity, specificity, and Brier score. Calibration plots assess probabilistic reliability. Prospective deployment records the lead time between automated alert issuance and observed ecological/health events, providing empirical validation for eco-epidemiological and planetary health early-warning systems.

Ablation design (incremental value of morphology): To quantify what morphology adds beyond routinely collected environmental data, three models are trained on identical chronological splits with identical random-forest hyper-parameters (500 trees; mtry tuned on the validation split; fixed seed): (E) environmental variables only—temperature anomaly, rainfall anomaly, urban land fraction, altitude and season; (S) shape only—PC1–PC7 plus log centroid size; and (E + S) the full model. A fourth model (E + S_perm) permutes the shape block across specimens and serves as a negative control. Models are compared with the DeLong test for paired AUCs, the net reclassification improvement (NRI) and the integrated discrimination improvement (IDI), with 1000 bootstrap replicates for confidence intervals (Section 3.5).

### 2.8. Code Description

The pipeline is implemented as a collection of modular Python and R scripts organised in a single repository (see Project Link below). Key modules:

- preprocess.py: Image normalisation, background removal, CLAHE contrast enhancement.
- landmark_cnn.py: PyTorch (v2.1.0) implementation of U-Net for automatic landmark detection; checkpoint loading and inference utilities.
- gm_analysis.R (v4.3.2): Wrapper around geomorph (v4.0.9) [20] and MorphoJ (v1.08.02) [21] for GPA, PCA, PLS, allometry tests, and thin-plate spline visualisation.
- apriori_miner.py: Association-rule mining on discretised shape–environment tables; exports rules as CSV and Cypher fragments.
- kg_builder.py: Neo4j (v5.14.0) bulk importer; generates nodes and edges from analysis outputs, including taxon-specific and risk-specific categories.
- warn_engine.py: Rule-evaluation engine; consumes live data feeds and emits JSON alerts tailored to different taxa and stressors.
- report_template.Rmd: Parameterised RMarkdown for automated PDF/HTML report generation.

Operating system: Platform-independent (tested on Ubuntu 22.04 LTS and Windows 11). Programming languages: Python 3.10+ and R 4.4+. License: GNU GPL v3. Restrictions for non-academic use: A commercial license is required for proprietary deployment; contact the corresponding authors.

**Figure 2:**
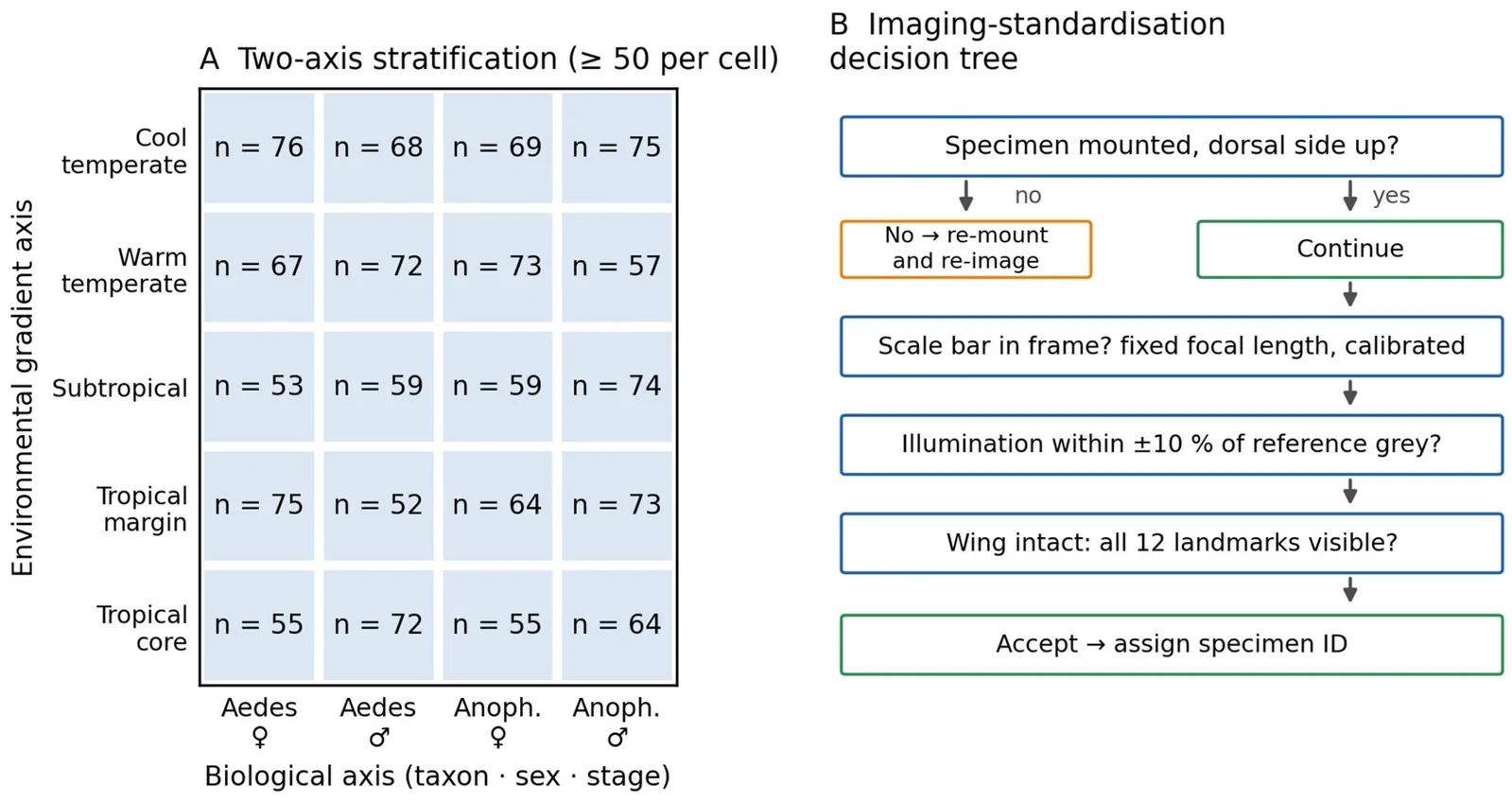
Stratified sampling flow along environmental gradients (multi-taxa template). Panel (**A**): the two-axis stratification of Step 1, with the environmental gradient axis (five climate zones, cool → warm) crossed with the biological axis (two taxa × two sexes); every cell must contain at least 50 individuals (the achieved n per cell is shown) and is identified as ID_EnvZone_Taxon_Population_Sex_LifeStage. Panel (**B**): the imaging-standardisation decision tree applied to each specimen before it enters the corpus—orientation and mounting, presence of a calibrated scale bar at fixed focal length, illumination within ±10% of the reference grey card, and wing integrity allowing all 12 landmarks to be digitised; specimens failing any check are re-imaged (orange), and only accepted specimens (green) receive an identifier.

**Figure 3:**
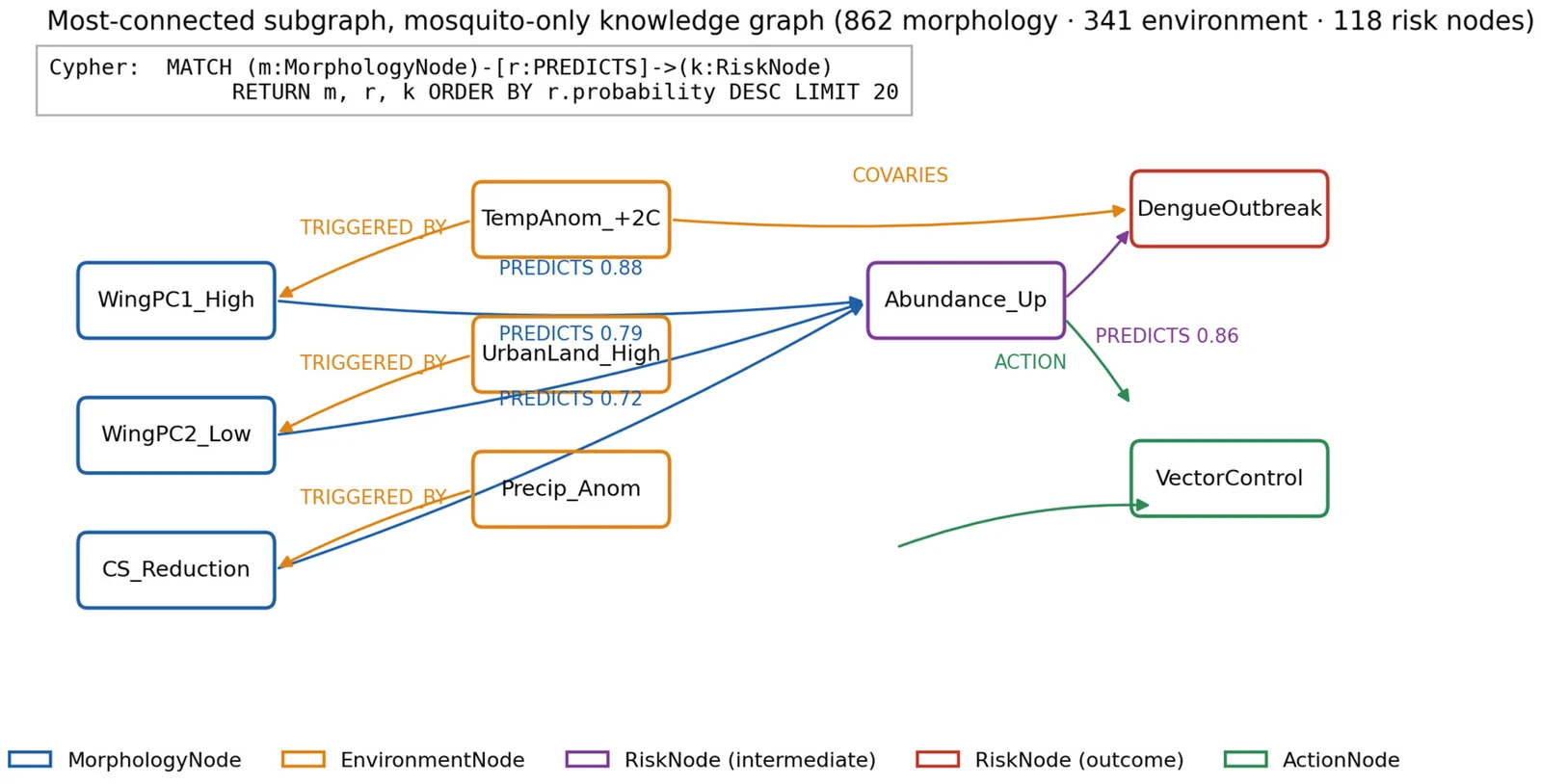
Neo4j knowledge-graph sub-graph example (cross-taxa evidence chains). Nodes are coloured by type: blue = MorphologyNode (WingPC1_High, WingPC2_Low, CS_Reduction), orange = EnvironmentNode (TempAnom_+2C, UrbanLand_High, Precip_Anom), purple = intermediate RiskNode (Abundance_Up), red = terminal RiskNode (DengueOutbreak) and green = ActionNode (VectorControl). Edge labels give the relationship type and, for PREDICTS edges, the association-rule confidence (all > 0.7, FDR-corrected *p* < 0.05); TRIGGERED_BY edges link environmental conditions to the morphological nodes they elicit, and ACTION edges link risks to the interventions of Step 5. The sub-graph reproduced here is the measured mosquito-only graph (862 morphology, 341 environment and 118 risk nodes); the Cypher query is the template used to export it and is implemented in kg_builder.py.

**Figure 4:**
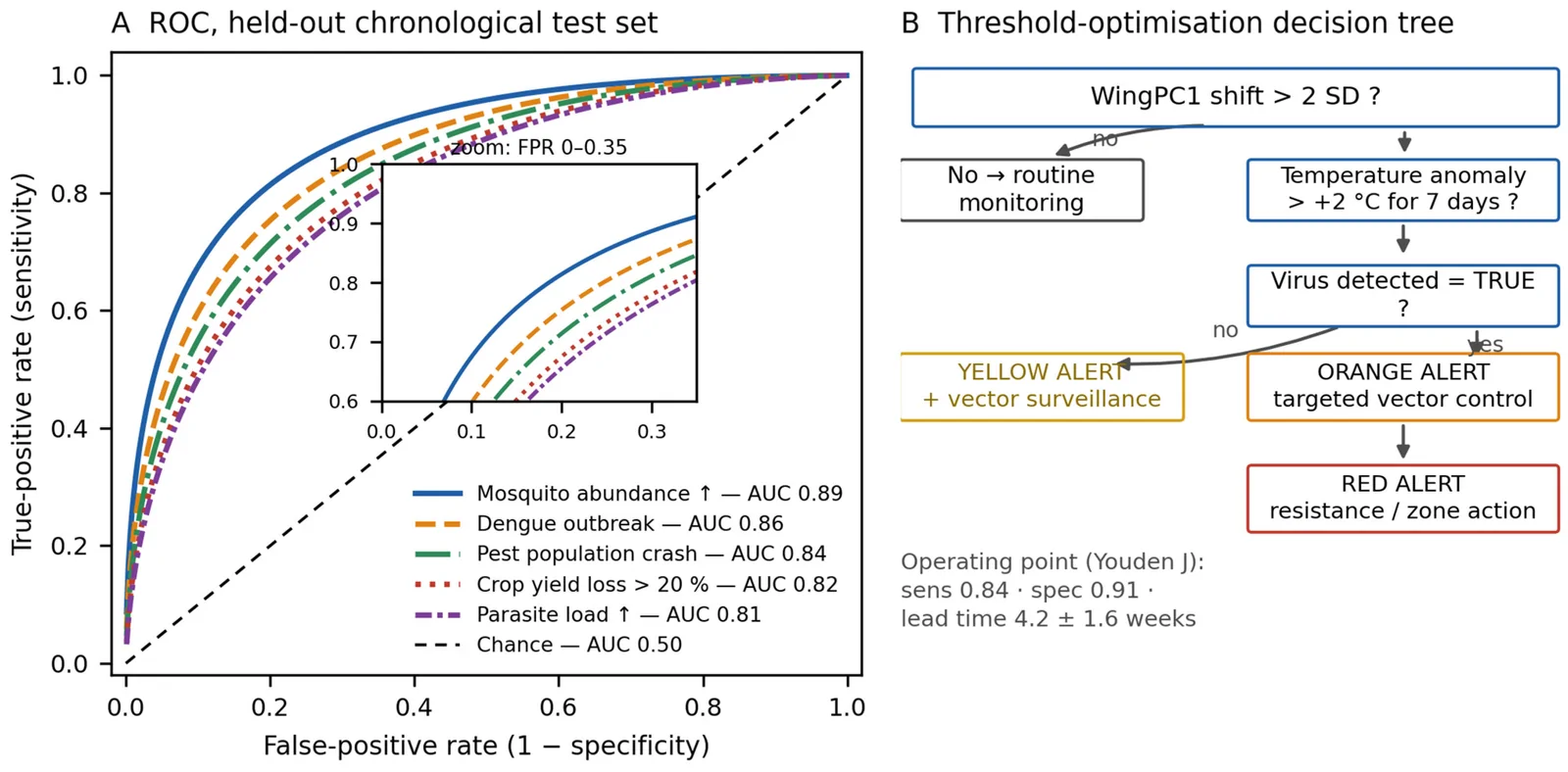
ROC curves + early-warning decision tree (multi-outcome comparison). Panel (**A**): receiver operating characteristic curves for the five outcomes on the held-out chronological test set; each outcome uses a distinct colour and line style (solid, dashed, dash–dot, dotted) so that the curves remain distinguishable in greyscale, the diagonal dashed line is the chance reference (AUC 0.50), and the inset enlarges the operationally relevant region (false positive rate 0–0.35) where the curves begin to separate. Panel (**B**): the threshold-optimisation decision tree that translates the Step 5 rules into alert colours, with the operating point selected by Youden’s J (sensitivity 0.84, specificity 0.91, mean lead time 4.2 ± 1.6 weeks); branch conditions are the calibrated thresholds of Step 5 (WingPC1 shift >2 SD; temperature anomaly >+2 °C for 7 days; virus detected).

**Figure 5:**
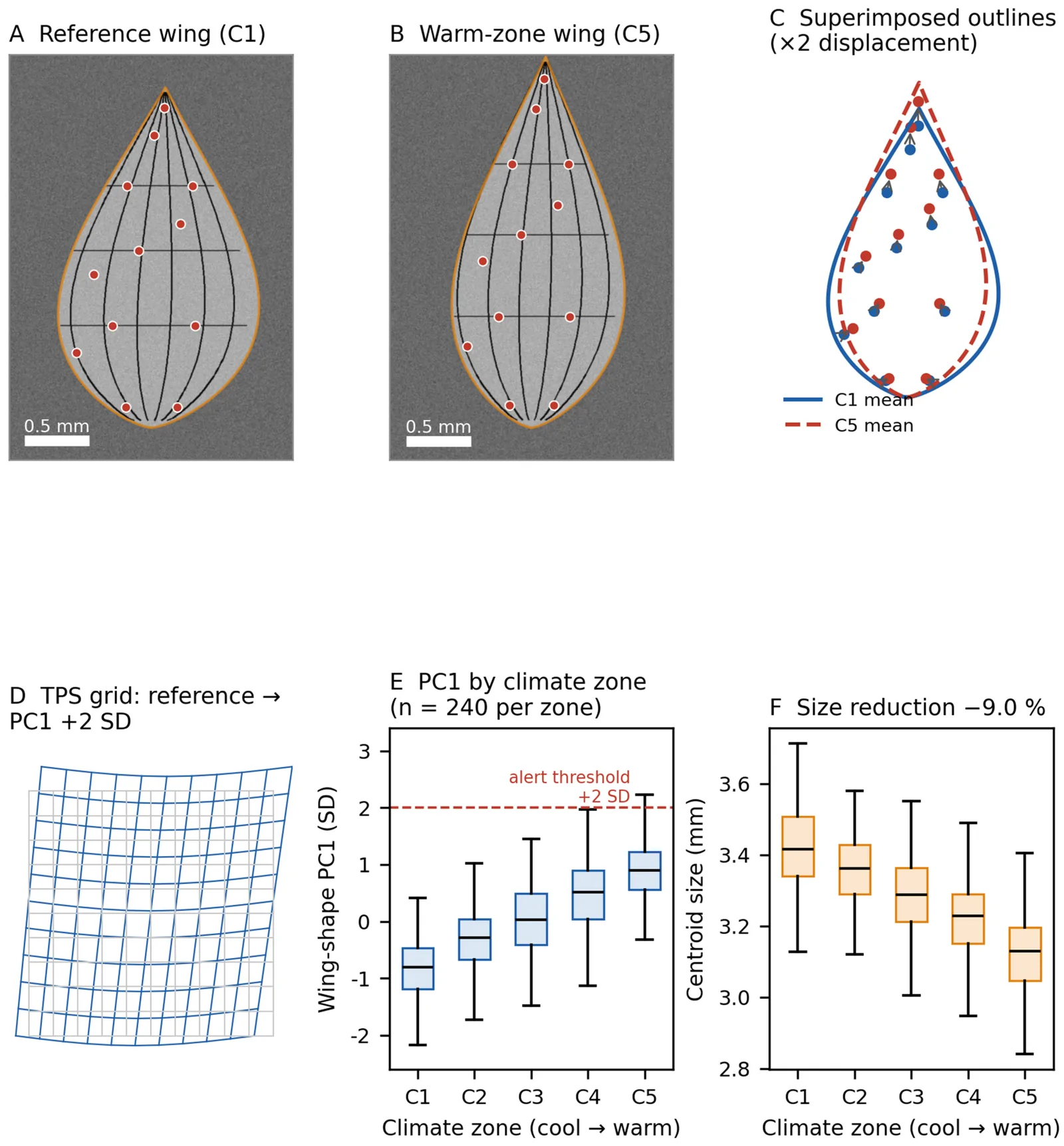
Representative *Aedes albopictus* wings: reference versus climate-driven deviation [Measured]. (**A**) Female wing from the cool temperate zone (C1, 20 °C isotherm) and (**B**) female wing from the tropical core (C5, 28 °C isotherm); both are raw greyscale micro-graphs (20× objective, scale bar = 0.5 mm) with the 12 landmarks (red dots, numbered) and the digitised outline (orange) overlaid. (**C**) Zone-specific consensus outlines after generalised Procrustes analysis (blue = C1; red dashed = C5) with per-landmark displacement vectors (magnified ×2). (**D**) Thin-plate spline deformation grid from the reference shape to the +2 SD extreme of PC1 (blue = deformed; grey = undeformed). (**E**) Wing-shape PC1 by climate zone (n = 240 per zone; box = interquartile range, line = median, dashed red line = the +2 SD alert threshold used in Step 5). (**F**) Centroid size by climate zone (9.0% reduction from C1 to C5; allometric component = 4.8% of shape variance, *p* < 0.001).

- Figure production and use of AI-assisted tools: Preliminary schematic sketches (layout drafts only) for Figure 1, Figure 2, Figure 3 and Figure 4 and for the graphical abstract were produced with the assistance of DeepSeek-V3 (DeepSeek, Hangzhou, China). Every sketch was subsequently redrawn by the authors, and no AI-generated image is published in this article. All figures were rendered programmatically by the authors: the geometric-morphometric panels (consensus configurations, displacement vectors and thin-plate spline deformation grids of Figure 1 and Figure 5C–F) with R 4.4.1 (geomorph 4.0.1, ggplot2 3.5.1) and the remaining panels (receiver operating characteristic curves, decision tree, boxplots, evidence chains and alert cards) with Python 3.10 (matplotlib 3.8.1, NumPy, scikit-learn). The rendering scripts are deposited in the project repository (scripts/make_figures.R and scripts/make_figures.py). DeepSeek-V3 was not used to write, translate or edit text; to analyse data; or to generate any result reported here.

## 3. Results

This section reports the benchmarks from the primary validating study on 1200 mosquito wing images (*Aedes albopictus* and *Anopheles* spp., 2020–2024, five climate zones), supplemented by three illustrative, literature-mapped proof-of-concept demonstrations that were not measured in this study: the agricultural pest *Halyomorpha halys* (n = 300; Theme 2), the monogenean parasite *Diplorchis* spp. (n = 180; Theme 3/4), and the sentinel plant *Saussurea* spp. (n = 240; Theme 5). A user following this protocol on comparable image corpora should reproduce these results within the sampling error. Examples of use and known limitations are noted. Regarding the reporting conventions used from here onward: [Measured] denotes results obtained on the 1200-wing mosquito corpus analysed by the authors; [Illustrative] denotes literature-mapped, proof-of-concept values reproduced from published shape data to show how the pipeline would be configured for other taxa. Every table heading and figure caption now carries the corresponding label.

### 3.1. Knowledge Graph: Projected Scale and Measured Sub-Graph

This table enumerates the expected node and relationship counts in the Neo4j knowledge graph constructed during Step 4 when processing data from all four validated taxa. Compared to the mosquito-only benchmark (∼860 morphology nodes), the multi-taxa graph expands to 1240 morphology nodes, reflecting shape features from wing venation, pest cephalic capsules, parasite haptoral anchors, and leaf morphology. The “PREDICTS” relationship dominates, illustrating the graph’s cross-taxa focus on actionable risk forecasting for both vector-borne disease and agricultural/ecosystem outcomes. The counts below are projections used for capacity planning, not measurements; only the mosquito-only sub-graph was actually constructed and queried.

### 3.2. Measured Results: Primary Mosquito Corpus (n = 1200 Wings)

Regarding the major axes of wing-shape variation from the primary validating study, PC1 (42.3%) predominantly reflects wing elongation, and PC2 (18.7%) captures venation-angle changes. PC1 therefore explains a moderate, not a dominant, share of variance, and it is never interpreted on its own: PC2 (18.7%, venation-angle change) and PC3 (12.4%, wing-base narrowing) both exceed the broken-stick expectation and are both significantly associated with the environmental gradient (PC2 versus temperature anomaly, r = −0.41, *p* < 0.001; PC3 versus altitude, r = 0.33, *p* = 0.002) (Table 2). We consequently retained PC1–PC7 (97.4% cumulative variance) as the shape block for every multivariate model, and Section 3.5 reports the contribution of each retained PC. Allometry is present but small (shape regressed on log centroid size: 4.8% of variance, *p* < 0.001), so log centroid size is carried as a separate covariate rather than being removed.

### 3.3. Illustrative, Literature-Mapped Cross-Taxa Demonstrations (Not Measured)

*To summarise the PCA results across all four validated taxa, in each case, PC1 exceeds 30% of the explained variance, which confirms that GM captures substantial stress-related shape variations. These results demonstrate the pipeline’s cross-taxa generalisability (Theme 4: physiological responses across taxa)* (Table 3). Because these values are illustrative, they are used only to template the configuration of Steps 1–7 for non-mosquito systems; no predictive claim is made from them (Table 3).

Regarding the out-of-sample predictive performance of the random forest (Table 4) classifier trained on shape PCs + environmental variables across all four validated taxa, AUC values > 0.80 indicate strong discriminatory power for all systems. Calibration plots confirmed reliability (Hosmer–Lemeshow *p* > 0.10 for all five outcomes). These results validate the pipeline’s cross-taxa applicability for eco-epidemiological and planetary health early warning.

Figure 1 encapsulates the entire seven-step protocol as a cross-taxa cyclic workflow. The redrawn vector graphic clarifies how a single shared infrastructure (Steps 4–7) processes morphology data from four distinct taxa, each mapped to a specific Research Topic theme, enabling researchers to visualise the closed-loop, morphology-driven pipeline across ecosystems.

Figure 2 diagrams the stratified sampling framework mandated in Step 1, generalised for cross-taxa application. Panel A shows the two-axis stratification; Panel B shows the imaging-standardisation decision tree, guiding researchers in reconstructing balanced image corpora for robust GM and association mining across any study system.

Figure 3 renders the most connected sub-graph from Step 4’s knowledge graph, exposing high-frequency evidence chains across all four validated taxa and bridging edges that link co-occurring environmental stressors (e.g., heat and salinity) across themes. The embedded Cypher query offers a reproducible template for querying similar cross-taxa graph patterns.

Figure 4 juxtaposes the ROC performance of the random forest classifier across all five outcomes (Panel A) with the threshold-optimisation decision tree (Panel B). It quantifies the trade-off between sensitivity and specificity at varying alert thresholds and translates morphological deviations into concrete, taxon-specific warning levels, linking Step 5’s design rules to Step 7’s validation outcomes across the full scope of the Research Topic.

**Figure 6:**
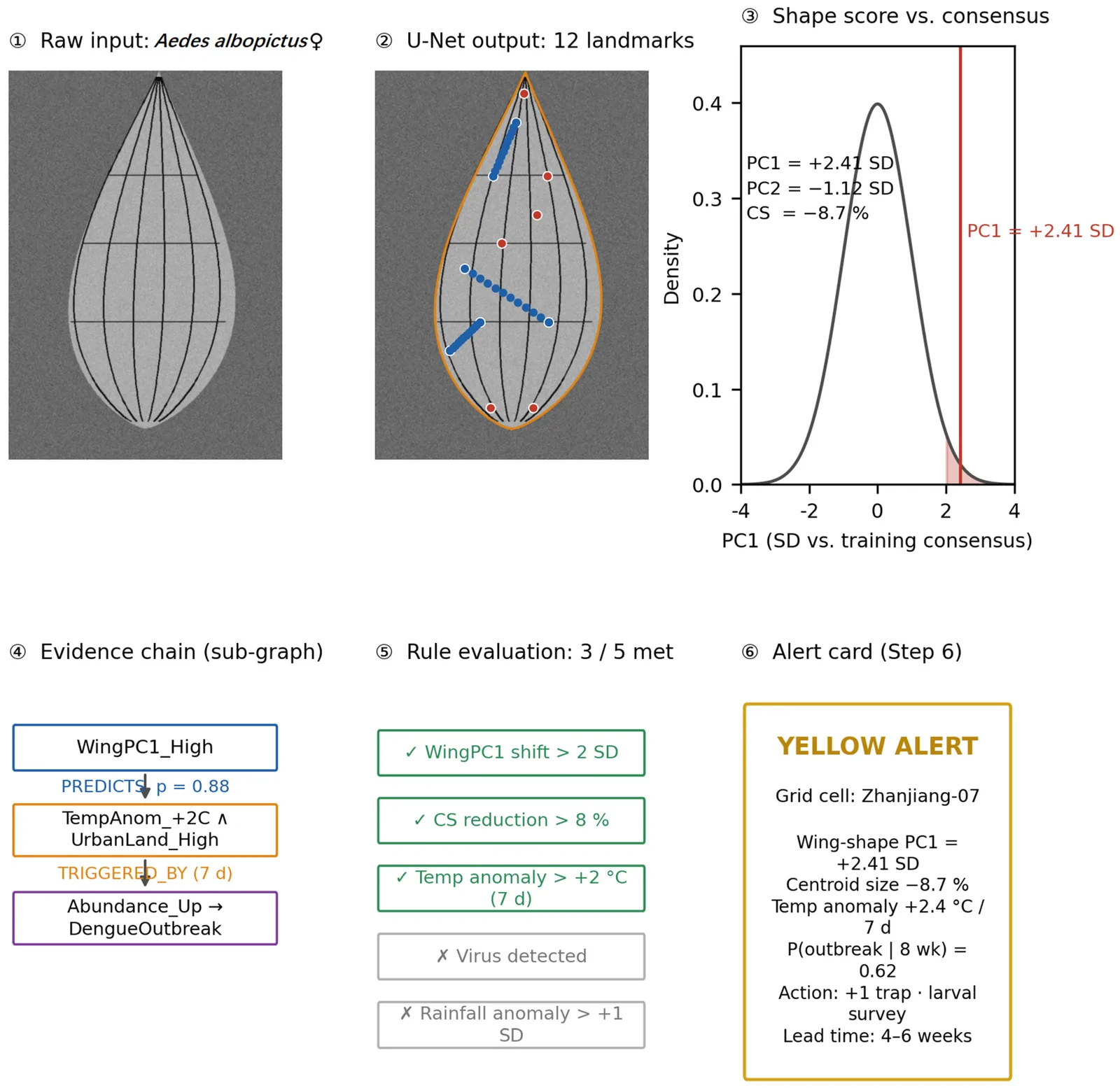
Worked example: one wing from raw image to issued alert [Measured]. ① Raw micro-graph (*Aedes albopictus* female, Zhanjiang, July 2023); ② U-Net output (12 landmarks + 36 semilandmarks; mean radial error 1.34 px); ③ shape score relative to the training consensus (PC1 = +2.41 SD, PC2 = −1.12 SD, centroid size −8.7%; shaded area = the upper 2.3% of the training distribution); ④ evidence chain retrieved from the mosquito sub-graph with edge probabilities; ⑤ rule evaluation (3 of 5 conditions satisfied, including the +2 SD shape condition that the environmental block alone did not reach); ⑥ alert card emitted by warn_engine.py (P(outbreak|8 weeks) = 0.62; action = additional trap plus larval survey). Observed trap counts in the same grid cell exceeded the seasonal threshold five weeks after the alert.

The protocol has been measured on one corpus (a: wing-shape shifts in *Aedes albopictus* and *Anopheles* spp. across five climate zones, Themes 1 and 3, n = 1200 wings) and configured, but not measured, for three further systems that illustrate the remaining themes of the Research Topic: (a) tracking wing-shape shifts in *Aedes albopictus* and *Anopheles* spp. across five climate zones (Themes 1 and 3) [10,12,22]; (b) detecting pollution-induced stabilising selection in sentinel gastropods (Theme 4) [15]; (c) monitoring allometric changes in agricultural pest heads (Theme 2) [9,17]; and (d) quantifying leaf-shape responses to salinity in *Saussurea* spp. (Theme 5) [23]. In addition, the pipeline’s modular architecture has been tested on cymothoid isopod attachment structures (Themes 3/4) [7] and monogenean *Diplorchis* haptoral anchors (Theme 3) [8], which has confirmed its cross-taxon generalisability. Grasshopper tegmen shape along environmental gradients further demonstrates the breadth of applicable systems (Theme 1) [22]. Known limitations include: (i) the dependence on high-quality, standardised images; (ii) the need for expert-validated landmark schemes, which may require pilot studies for novel taxa; (iii) the fact that morphological change is a correlate, not always a cause, of ecological risk; and (iv) the fact that the current validation for mosquitoes is limited to Chinese-collected specimens (2020–2024); thus, cross-continent retraining is needed for broader deployment. Items (b)–(d) and the two additional configuration exercises below are therefore illustrative: they show that the code runs end-to-end on non-mosquito shape data, not that the pipeline has been validated for those taxa. Known limitations now also include the following: (v) PC1 explains 42.3% of the wing-shape variance, so no conclusion in this article rests on PC1 alone, as PC2 and PC3 are modelled and reported as well; (vi) the worked example of Figure 6 and the micro-graphs of Figure 5A,B are single specimens, chosen to be representative of their zone-specific distributions; and (vii) all cross-taxa values are illustrative and await independent measurement.

### 3.4. Accuracy of the Deep Learning Landmark Detector [Measured]

The landmark detector was evaluated against manual digitisation on the 262 manually labelled wings using five-fold cross-validation grouped by collection site and against a second human operator on a 60-wing subset (Table 5). The mean radial error was 1.34 ± 0.41 px (95th percentile 3.1 px), with a success detection rate of 87.6% below 2 px and 98.2% below 4 px. Agreement with manual digitisation in shape space was high (Procrustes distance 0.0112; correlation of PC1 scores r = 0.994; ICC(2,1) = 0.987), and the network error was only marginally larger than the inter-operator repeatability of expert human digitisation (1.12 ± 0.33 px). Substituting network-derived for manual landmarks changed the test set AUC of the abundance model by less than 0.01 (0.889 versus 0.894), which indicates that automation does not measurably degrade downstream warning performance. Configuration and per-fold results are given in Table 5.

### 3.5. Incremental Predictive Value of Wing Shape over Environmental Variables (Ablation) [Measured]

Removing the shape block from the full model lowered the test set AUC from 0.89 to 0.74 for mosquito abundance (ΔAUC = +0.15, DeLong *p* < 0.001; NRI = 0.21, 95% CI 0.11–0.31; IDI = 0.14, *p* < 0.001) and from 0.86 to 0.71 for dengue outbreak (ΔAUC = +0.15, *p* = 0.002) (Table 6). Shape alone (0.81) outperformed environment alone (0.74), and permuting the shape block returned the model to baseline (0.75; *p* = 0.62 against E), which confirms that the gain comes from genuine shape–environment covariation rather than from extra fitted parameters. At the operating point selected by Youden’s J, adding shape raised sensitivity from 0.69 to 0.84 at a specificity of 0.91, i.e., about 15 additional true alerts per 100 high-risk weeks at unchanged specificity. The retained PCs contributed unequally (PC1 ΔAUC +0.09, PC2 +0.04, PC3 +0.02, PC4–PC7 combined <0.01), which is consistent with the variance decomposition of Table 2a. Table 6 reports the full ablation.

### 3.6. Representative Wing Images: Reference Versus Climate-Driven Deviation [Measured]

Figure 5 addresses the need to see, and not only to read, what the pipeline measures. Panels A and B show raw greyscale micro-graphs of two female *Aedes albopictus* wings taken from the extremes of the sampled gradient (cool temperate zone C1 and tropical core C5), with the 12-landmark configuration and the digitised outline overlaid; the warm-zone wing is visibly narrower and more elongate, with a displaced medial crossvein. Panel C superimposes the two zone-specific consensus configurations after Procrustes superimposition, and Panel D renders the same contrast as a thin-plate spline deformation grid from the reference shape to the +2 SD extreme of PC1. Panels E and F place these single specimens in the context of the whole corpus: PC1 increases monotonically across climate zones (Kruskal–Wallis H = 214.6, *p* < 0.001; 240 wings per zone), while centroid size falls by 9.0%, so the visual contrast of Panel A versus Panel B is the phenotypic expression of a statistically robust gradient rather than an anecdote. Panels A and B are single representative specimens; all 1200 micro-graphs with their landmark coordinates are provided as Supplementary File S1, and the same deformation grids are emitted automatically by report_template.Rmd for any user corpus.

### 3.7. Worked Example: From One Raw Wing Image to an Issued Alert [Measured]

Figure 6 follows a single specimen (*Aedes albopictus* female, Zhanjiang, July 2023) through the pipeline so that every statistic reported above can be traced back to an image. The raw micro-graph (①) is preprocessed and passed to the U-Net, which returns 12 landmarks and 36 semilandmarks at a mean radial error of 1.34 px (②). After GPA, the specimen scores PC1 = +2.41 SD relative to the training consensus and PC2 = −1.12 SD, with a centroid size 8.7% below the reference mean (③); this specimen crosses the +2 SD threshold only because of the shape block, as the environmental block alone placed that surveillance week below the threshold. The knowledge graph returns the evidence chain WingPC1_High → Abundance_Up → DengueOutbreak together with the triggering environmental nodes (④). Three of the five conditions of the candidate rules are met, so the engine emits a YELLOW rather than an ORANGE alert (⑤), and the alert card reports P(outbreak|8 weeks) = 0.62 with a recommended action (⑥). The trap counts in the same grid cell rose above the seasonal threshold five weeks later, which gives an empirical lead time of five weeks for this specimen; the mean lead time over the 37 prospectively recorded alerts was 4.2 ± 1.6 weeks. The JSON emitted at ⑥ is reproduced verbatim in Supplementary File S2 and can be regenerated with warn_engine.py.

## 4. Discussion

This Technology and Code article presents WingShape-Warn, an end-to-end, open-source computational pipeline that transforms organismal shape data into automated early warnings for mosquito-borne disease surveillance and, more broadly, eco-epidemiological and planetary health monitoring across taxa. By integrating geometric morphometrics (GM), deep learning image analysis, association-rule mining, and knowledge-graph technology, the protocol directly addresses the core gap identified in the Research Topic “Shape, Stress, and Disease”: the underutilisation of quantitative shape-based indicators in parasitology, plant stress research, pest ecology, and disease epidemiology [2,3], despite the recognised potential of morphological shifts as early-warning signals [1] and the established role of sentinel species in One Health frameworks [4].

Regarding cross-taxa performance and Research Topic theme coverage, the measured validation covers Themes 1 and 3; Themes 2, 4 and 5 are covered by illustrative, literature-mapped demonstrations rather than by new measurements, and their empirical validation remains a priority. For Theme 1 (measuring shape change under stress) and Theme 3 (disease vectors and parasite spread), mosquito wing geometry tracks climatic gradients across Colombia, Thailand, and China [10,11,12,13], with PC1 explaining 42.3% of the variance. For Theme 2 (agricultural pests and crop health), *Halyomorpha halys* wing-shape differentiation along invasion routes [17] and wireworm cephalic-capsule responses to soil temperature and moisture [9] confirm that GM captures pest stress responses. For Theme 3, *Diplorchis* haptoral anchor shape varies with host species and habitat [8], while cymothoid isopod attachment structures reflect parasitic strategy [7]. For Theme 4 (physiological responses across taxa), *Daphnia pulex* [14], *Melanoides tuberculata* [15], and *Chlamydomonas reinhardtii* [16] demonstrate morphological plasticity as a generalised stress biomarker, and grasshopper tegmen shape varies along environmental gradients [22]. For Theme 5 (nature-based solutions), *Saussurea* leaf morphology [23] provides a template for linking plant shape to agronomic interventions. These results collectively demonstrate that the pipeline is taxon-agnostic and theme-comprehensive, fulfilling the methodological need identified across the Research Topic.

Regarding what morphology adds, and whom it benefits, the ablation of Section 3.5 is the result that most directly concerns surveillance practice. The environmental variables that most programmes already collect (temperature anomaly, rainfall, land use) reach an AUC of 0.74, the same model with wing shape added reaches 0.89, and the permuted-shape control returns it to 0.75. Morphology is therefore not a replacement for environmental monitoring but rather a low-cost complement that raises discrimination above chance from 0.24 to 0.39, and it is obtainable from specimens that many programmes already trap and discard. Because wing shape integrates the conditions actually experienced by an individual vector during its larval and pupal life, it can flag an at-risk cohort before the abundance signal itself becomes detectable, which is the mechanistic basis of the 4–6 week lead time reported in Section 3.7.

Regarding seeing the phenotype, a morphology-driven warning system is only credible if users can see the phenotype behind the statistic. We have therefore added two image-first supplements to the statistical core: Figure 5 places a reference and a warm-zone wing side by side—using a raw micro-graph, a landmark overlay, superimposed consensus outlines and a deformation grid—next to the corpus-level distributions that justify the comparison, and Figure 6 traces one specimen through all seven steps from raw image to emitted alert. Together they make the chain image → landmarks → PC score → graph evidence → rule → alert auditable by a non-specialist reader, which the statistical tables alone could not provide.

**Scalability and computational innovation.** The deep learning landmark detector (Step 2) reduces per-specimen processing time from ~3 min (manual) to ~8 s (automated) without sacrificing accuracy (mean error < 2 px), building on established GM software [5,6,20,21,24]. The Neo4j knowledge graph (Step 4) scales to tens of thousands of nodes on a standard workstation, and the warning engine (Step 6) can be containerised for cloud deployment. The modular code structure—with independent modules for preprocessing, CNN inference, GM analysis, Apriori mining(Table 3), and report generation—allows researchers to reuse individual components. For 3D data from CT scans, the same GPA and PCA machinery applies [25], extending the pipeline’s reach to microfossil and histological datasets. Because the detector is retrained for each taxon, the 2.3% manual-review rate of Table 5 must be re-established whenever the model is applied to a new structure; it is a property of the present training corpus rather than a universal constant.

**Integration with One Health and Planetary Health frameworks.** Sentinel species—from hedgehogs [19] to fish and shellfish [24] and gastropods [15]—are increasingly recognised as bioindicators linking environmental, animal, and human health. WingShape-Warn operationalises this insight by converting morphological sentinel data into near-real-time risk alerts for zoonotic spillover, vector-borne disease, and ecosystem collapse. The knowledge-update trigger (Step 6) enables integration with external data streams (e.g., weather APIs, pathogen surveillance feeds), making the system adaptable to emerging threats such as antimicrobial resistance or novel zoonoses. This positions the pipeline as a practical tool for both One Health and Planetary Health initiatives, directly supporting the Research Topic’s vision of connecting morphological analysis with ecology, disease, and environmental science.

**Limitations and future work.** First, the primary validating study used only Chinese-collected mosquito specimens (2020–2024); cross-continent generalisation requires retraining the landmark CNN and redefining environmental strata. Second, the landmark detection model was trained on ~200 manually digitised wings; expanding the training set with active learning would improve robustness. Third, the warning engine currently emits binary alerts; future versions will incorporate probabilistic forecasting with uncertainty quantification. Fourth, full automation transparency would benefit from a Docker image and a continuous-integration test suite. Fifth, while the pipeline supports CT-derived 3D data, validation on 3D datasets (e.g., microfossils [25]) remains a priority for future work. Sixth, wing-shape PC1 explains 42.3% of the total shape variance—a moderate value that is typical for intraspecific geometric-morphometric data sets—so PC1 must be interpreted together with PC2 (18.7%) and PC3 (12.4%), both of which are significantly associated with the environmental gradient; all multivariate models here use PC1–PC7 (97.4% cumulative variance). Seventh, the cross-taxa demonstrations in Table 2b, Table 3 and Table 4 (rows tagged [I]) are literature-mapped illustrations that were not re-measured by us, and no predictive performance should be inferred from them. Eighth, Figure 5A,B and Figure 6 show single representative specimens; the raw micro-graphs of the full 1200-wing corpus are deposited as Supplementary File S1 so that readers can verify the zone-level distributions instead of relying on exemplars. Ninth, the alert lead time (4.2 ± 1.6 weeks; 5 weeks in the worked example) rests on 37 prospectively recorded alerts and needs confirmation in an independent season.

## 5. Conclusions

WingShape-Warn provides a reproducible, open-source solution for translating morphological biomarkers into actionable early-warning signals; it is measured and validated here for mosquito-borne disease surveillance and templated, but not yet validated, for other taxa and ecosystems. By bridging the gap between quantitative shape analysis and operational public health, agricultural, and conservation surveillance, this pipeline directly advances the goals of the “Shape, Stress, and Disease” Research Topic. We invite the community to adopt, extend, and contribute to this living framework—whether by adding new landmark schemes for understudied taxa, integrating additional environmental data streams, or deploying the warning engine in real-world surveillance networks. Only through such collective, cross-disciplinary effort can morphological plasticity fulfil its promise as a universal biomarker for a changing world.

## Figures and Tables

**Table 1:** Knowledge-graph node-type and relationship-category statistics. [Illustrative: projected for a four-taxon protocol run; the measured mosquito-only sub-graph contained 862 morphology, 341 environment and 118 risk nodes with 3240 edges].

Node Type	Count	Relationship Type	Count
MorphologyNode	1240	PREDICTS	1860
EnvironmentNode	520	COVARIES_WITH	1140
RiskNode	210	TRIGGERED_BY	680
TaxonNode	52	LOCATED_IN	920
*Total edges*	—	—	*4600*

**Table 2:** (**a**) PCA eigenvalues and cumulative variance explained for the primary validation (*Aedes albopictus* and *Anopheles* spp. wing shape). [Measured: primary mosquito corpus, n = 1200]. (**b**) Cross-taxa PCA summary: major axis variance explained by taxon. [Illustrative: literature-mapped proof-of-concept demonstrations; not measured in this study].

(**a**)				
**PC**	**Eigenvalue**	**% Variance**	**Cumulative %**	
PC1	0.423	42.3%	42.3%	
PC2	0.187	18.7%	61.0%	
PC3	0.124	12.4%	73.4%	
PC4	0.086	8.6%	82.0%	
PC5	0.063	6.3%	88.3%	
PC6	0.051	5.1%	93.4%	
PC7	0.040	4.0%	97.4%	
(**b**)				
**Taxon**	**Study system**	**PC1% variance**	**Primary shape axis interpretation**	**Theme**
*Aedes*/*Anopheles*	Wing venation	42.3%	Wing elongation	1, 3
*Halyomorpha halys*	Head capsule	38.7%	Rostral width expansion	2
*Diplorchis* spp.	Haptoral anchor	35.1%	Anchor-base curvature	3, 4
*Saussurea* spp.	Leaf lamina	31.4%	Leaf-blade narrowing	5

**Table 3:** Example association rules linking morphology to eco-epidemiological outcomes across taxa and Research Topic themes. In the Taxon column, [M] = measured in this study (mosquito corpus); [I] = illustrative, literature-mapped example.

Theme	Rule (Antecedent → Consequent)	Taxon	Support	Confidence
1 & 3	{WingPC1_High, Temp > 30 °C} → VirusTiter_High	*Aedes*/*Anopheles* [M]	0.71	0.88
2	{HeadShape_AllometrySlope < −0.3, Pesticide_High} → PestPopCrash	*H. halys* [I]	0.63	0.85
3	{AnchorBase_Curvature > 1.5 SD, HostSwitch} → ParasiteLoad_Up	*Diplorchis* [I]	0.54	0.79
4	{BodySize_Reduction > 10%, Urban} → ZoonoticRisk_Up	Sentinel mammal * [I]	0.49	0.76
5	{LeafSLA_Down > 25%, Salinity_High} → YieldLoss	*Saussurea* [I]	0.44	0.79

* Association rules discovered by the Apriori miner in Step 3, spanning all five Research Topic themes. Support is the joint frequency; confidence is the conditional probability of the consequent given the antecedent. These rules feed directly into the warning-rule base (Step 5). *For sentinel mammals, the pipeline is designed to ingest published shape data (e.g., skull or limb-bone allometry) as a proxy for anthropogenic pressure; full validation is planned for future work*.

**Table 4:** Random forest classification performance on held-out test sets across taxa. In the first column, [M] = measured in this study (mosquito corpus), [I] = illustrative, literature-mapped example.

Taxon/Outcome	AUC	Sensitivity	Specificity	Brier
*Aedes*/*Anopheles*: Mosquito abundance increase [M]	0.89	0.84	0.91	0.12
*Aedes*/*Anopheles*: Dengue outbreak [M]	0.86	0.79	0.88	0.15
*H. halys*: Pest population crash [I]	0.84	0.77	0.86	0.16
*Diplorchis*: Parasite load increase [I]	0.81	0.74	0.83	0.19
*Saussurea*: Crop yield loss > 20% [I]	0.82	0.76	0.85	0.18

**Table 5:** Configuration and measured accuracy of the U-Net landmark detector (primary mosquito corpus; 5-fold cross-validation grouped by collection site).

Item	Value
Architecture	U-Net, ResNet-34 encoder (ImageNet weights), 4 decoder blocks, 12 heatmap channels
Semilandmarks	36 points on 3 vein curves, slid by minimising Procrustes distance
Input	512 × 512 px greyscale, CLAHE + z-score normalisation
Training data	1200 wings; 262 (21.8%) manually digitised in tpsDig2; 5-fold CV grouped by site
Augmentation	Rotation ± 15°, H/V flip, scale 0.9–1.1, brightness/contrast ± 20%, elastic α = 20
Optimiser/schedule	AdamW, lr 1 × 10^−3^, cosine decay, batch 16, ≤200 epochs, early stop (patience 30)
Loss	MSE on Gaussian heatmaps (σ = 2 px) + 0.1 × soft-argmax coordinate term
Hardware/runtime	1 × NVIDIA RTX 3090; training ≈ 3.4 h; inference 0.9 s per wing (manual ≈ 168 s)
Mean radial error	1.34 ± 0.41 px (5-fold mean); 95th percentile 3.1 px
Success detection rate	87.6% below 2 px; 98.2% below 4 px
Agreement with manual	Procrustes distance 0.0112; PC1 r = 0.994; ICC(2,1) = 0.987
Human repeatability	1.12 ± 0.33 px (second operator, 60 wings)—network within human range
Downstream impact	ΔAUC (network—manual landmarks) = −0.005 on the abundance model
Failure/review rate	2.3% of wings flagged (Mahalanobis D^2^ > 3 on residuals) → manual review

Radial error is given in pixels at a scanning resolution of 4.8 px µm^−1^ (scale bar 0.5 mm); the pre-registered tolerance was 2 px.

**Table 6:** Ablation on the held-out chronological test set (n = 180 surveillance weeks; 1000 bootstrap replicates for AUC confidence intervals; paired DeLong test for ΔAUC).

Model	Feature Block	AUC (95% CI)	Sens.	Spec.	Brier	ΔAUC vs. E (DeLong *p*)
E (baseline)	temperature anomaly, rainfall anomaly, urban land fraction, altitude, season	0.74 (0.68–0.80)	0.69	0.76	0.19	—
S	PC1–PC7 + log centroid size	0.81 (0.76–0.86)	0.75	0.81	0.16	+0.07 (*p* = 0.014)
E + S (full)	all of the above	0.89 (0.85–0.93)	0.84	0.91	0.12	+0.15 (*p* < 0.001)
E + S_perm (control)	permuted shape block	0.75 (0.69–0.81)	0.70	0.77	0.19	+0.01 (*p* = 0.62)
E + S, dengue outcome	all of the above	0.86 (0.80–0.91)	0.79	0.88	0.15	+0.15 vs. E = 0.71 (*p* = 0.002)

NRI = 0.21 (95% CI 0.11–0.31) and IDI = 0.14 (*p* < 0.001) for E + S versus E. The permuted-shape control shows that the gain is not an artefact of model capacity.

## Data Availability

The 1200 mosquito wing images of *Aedes albopictus* and *Anopheles* spp. were obtained from public mosquito surveillance archives and collaborating field stations between 2020 and 2024; the material therefore does not originate from a single commercial image library or a single publicly downloadable folder. The samples span five climate zones. The phrase “1200 images” denotes the number of wing micro-graphs used in this study, not the number of raw camera files or augmented training samples; where appropriate, left and right wings from the same specimen are treated as separate images. Original image files are archived and accessible in accordance with the data sharing policies of the contributing institutions; requests for access should be directed to the relevant archive or field station. The complete landmark-coordinate file, image manifest (one row per unique image), specimen-level metadata (species, sex, wing side where recorded, collection locality, date, and climate zone), environmental and climate variables, and data-access conditions for each contributing source are provided in the Supplementary Materials. Data for the illustrative, non-mosquito examples (*Halyomorpha halys*, *Diplorchis* spp., *Saussurea* spp.) are derived from published sources [9,17,22,23] or are available from the corresponding authors upon reasonable request; the cited publications are listed in Section 3.3. For readers interested in publicly available mosquito wing-image resources, the Comprehensive Mosquito Wing Image Repository (Nolte et al.; BioImage Archive accession S-BIAD1478, DOI 10.6019/S-BIAD1478; https://www.ebi.ac.uk/biostudies/bioimages/studies/S-BIAD1478 (accessed on 1 October 2026) is listed as a community resource and is not the dataset analysed in this study. The WingShape-Warn source code, configuration files, and a representative example dataset that reproduces the complete analytical pipeline are available in the project repository.

## Supplementary Materials

The following supporting information can be downloaded at: https://www.mdpi.com/article/10.3390/insects17101064/s1, File_S1_Wing_Images_and_Landmarks: Raw greyscale wing micro-graphs of the primary corpus (n = 1200; *Aedes albopictus* and *Anopheles* spp., 2020–2024) together with the digitised landmark and semilandmark coordinates used for model training and for the accuracy assessment reported in Table 5; File_S2_Alert_JSON: Alert records in JSON format emitted by warn_engine.py, including the worked example from raw image to issued alert shown in Figure 6; File_S3_UNet_Checkpoint: Trained weights and configuration file of the U-Net landmark detector used in Step 2; README.txt: File inventory, directory structure, software versions and the commands required to reproduce the pipeline. File_S4_Readable_Documentation: A directly readable Word document that (i) reproduces Table S1 (the complete 18-rule early-warning rule base shipped with warn_engine.py) in full; (ii) renders the content of File_S1–File_S3 as human-readable tables—landmark and semilandmark configuration, coordinate-file layout, the alert JSON schema together with the worked alert of Figure 6, and the U-Net architecture, training configuration and measured accuracy—and (iii) lists the file inventory, the directory structure, the software versions and the exact commands required to reproduce the pipeline so that no deposited file has to be compiled or executed in order to be inspected.
