# Supplementary material for: WingShape-Warn: A Morphology-Driven Early-Warning Pipeline for Mosquito-Borne Disease Surveillance
Source: Insects. 2026 Oct 15;17(10):1064. doi: 10.3390/insects17101064 (PMC13705500; doi:10.3390/insects17101064)
Supplement: Supplementary file 1 [file insects-17-01064-s001.zip › WingShape-Warn_Supplementary_File_S4_Readable_Documentation.pdf]

Supplementary File S4

WingShape-Warn — Readable Technical Documentation of the Supplementary Material

Manuscript insects-4542304 · "WingShape-Warn: A Morphology-Driven Early-Warning Pipeline for Mosquito-Borne Disease Surveillance" · Pipeline version 1.0.0

Why this file exists. The three supplementary folders (File_S1_Wing_Images_and_Landmarks, File_S2_Alert_JSON, File_S3_UNet_Checkpoint) are machine-readable by design: images plus coordinate tables, JSON alert records, and a trained PyTorch checkpoint. That is what the pipeline needs, but it forces a reader to write and run code before the material can be inspected. This document is the directly readable layer that sits on top of the folders. It (i) reproduces Table S1 — the complete 18-rule early-warning rule base — in full, (ii) renders the content of every folder as ordinary tables that open in any word processor, and (iii) reproduces the file inventory, the directory structure, the software versions and the commands required to reproduce the pipeline (README.txt). Nothing in this document has to be compiled, imported or executed to be read.

How to read the labels. [M] = measured in this study on the mosquito corpus (n = 1,200 wings, Aedes albopictus and Anopheles spp., 2020–2024, five climate zones). [I] = illustrative, literature-mapped template shipped with the code for a non-mosquito system; it has not been evaluated on measured data. The same convention is used in the main text.

1. Table S1 — Complete early-warning rule base (n = 18)

This is the rule base shipped with warn_engine.py v1.0.0 and referenced in Section 2.5 of the manuscript ("the rule base shipped with the code contains 18 rules (Supplementary Table S1)"). Rules R01–R09 are parameterised on the measured mosquito corpus; rules R10–R18 are configuration templates that ship with the code for the agricultural-pest, parasite / zoonotic and sentinel-plant compartments of the "Shape, Stress and Disease" Research Topic. Any rule can be replaced or extended by a user-supplied rule expressed in the same JSON schema (see Section 4), so the base is extensible rather than closed.

Rule ID	Theme	System (taxon / compartment)	Morphological antecedent (threshold)	Environmental / contextual antecedent	Consequent → alert level and action	Support / Conf. / Lift	Status
R01_dengue_warmzone	1, 3	Aedes albopictus / Anopheles spp. (mosquito)	WingPC1 shift > 2 SD; centroid-size reduction > 8%	Temperature anomaly > +2 °C for 7 d; rainfall anomaly > +1 SD; virus detected in pool	3 of 5 conditions → YELLOW (add one ovitrap, larval survey within 200 m, re-sample in 14 d); ≥4 of 5 → ORANGE; 5 of 5 → RED	0.34 / 0.78 / 1.9	[M]
R02_wingpc1_virus	3	Mosquito	Mean WingPC1 shift > 2 SD	Virus detected = TRUE	ORANGE → initiate targeted vector control	— / — / —	[M]
R03_asymmetry_heat	1, 3	Mosquito	Fluctuating asymmetry > 0.05	Temperature anomaly > +2 °C for 7 d	YELLOW → increase vector surveillance	— / — / —	[M]
R04_wingpc1_titer	1, 3	Mosquito	WingPC1 > upper tertile	Temperature > 30 °C	{VirusTiter_High} → evidence node feeding R01 / R02 escalation	0.71 / 0.88 / —	[M]
R05_cs_reduction_urban	1, 3	Mosquito	Centroid-size reduction > 8%	UrbanLand_High	YELLOW → container and larval-habitat survey	— / — / —	[M]
R06_wingpc2_altitude	1, 3	Mosquito	WingPC2 (venation angle) shift < −1.5 SD	Altitude band > 1,000 m	YELLOW → re-stratify the trap grid	— / — / —	[M]
R07_wingpc3_	1,	Mosquito	WingPC3 (wing-	Rainfall	YELLOW → verify and	— / — /	[M

rainfall	3		base narrowing) shift > 1.5 SD	anomaly > +1 SD	treat breeding sites	—	]
R08_shape_outlier	1, 3	Any taxon (internal quality gate)	Mahalanobis $D^2 > 3$ from the training centroid, in shape space or in landmark residuals	— (no environmental antecedent)	TECHNICAL → route the specimen to manual review (2.3% of the corpus) and fire the knowledge-update trigger	— / — / —	[M]
R09_escalation_dengue	3	Mosquito	P(outbreak within 8 wk) ≥ 0.75 from the shape + environment model	Evidence-chain probability (WingPC1_High → Abundance_Up → DengueOutbreak) ≥ 0.80	RED → emergency vector control plus public notification	— / — / —	[M]
R10_pest_allometry	2	Halyomorpha halys (agricultural pest)	Head-shape allometry slope < −0.3	Pesticide resistance = TRUE	RED → rotate active ingredients	0.63 / 0.85 / —	[I]
R11_pest_invasion_front	2	Agricultural pest	Wing-shape PC1 shift > 2 SD	New invasion front = TRUE	ORANGE → delimiting survey	— / — / —	[I]
R12_pest_soil_stress	2	Wireworm cephalic capsule (agricultural pest)	Cephalic-capsule PC1 shift > 1.5 SD	Soil temperature / moisture anomaly > +1 SD	YELLOW → adjust monitoring intensity and sowing date	— / — / —	[I]
R13_parasite_haptor	3, 4	Diplorchis spp. (monogenean parasite)	Anchor-base curvature > 1.5 SD	Host switch = TRUE	ORANGE → host-population screening	0.54 / 0.79 / —	[I]
R14_parasite_zoonosis	4	Parasite / zoonotic system	Egg-shape change > threshold	Zoonotic risk = High	ZOONOTIC ALERT → enhanced surveillance at peri-urban interfaces	— / — / —	[I]
R15_bodysize_urban	4	Sentinel mammal	Body-size reduction > 10%	Urban habitat	YELLOW → parasite-load monitoring	0.49 / 0.76 / —	[I]
R16_isopod_attachment	3, 4	Cymothoid isopod	Attachment-structure PC1 shift > 2 SD	Host or habitat change	YELLOW → re-baseline the landmark scheme	— / — / —	[I]
R17_leaf_salinity	5	Saussurea spp. (sentinel plant)	Leaf SLA reduction > 25%	Soil salinity > threshold	AGRONOMIC ADVISORY → irrigation adjustment, salt-tolerant varieties	0.44 / 0.79 / —	[I]
R18_leaf_pollution	5	Sentinel plant	Leaf-margin shape PC1 shift > 2 SD	Pollution index = High	YELLOW → bioindicator re-sampling	— / — / —	[I]

Table S1. Early-warning rule base shipped with warn_engine.py v1.0.0 (n = 18 rules). Reproduced here in full so that the rule base can be read without executing any code.

Support, confidence and lift are reported only for rules retained by the Apriori miner of Step 3 (min support 0.3, min confidence 0.7, lift ≥ 1.2, χ^2 test with Benjamini–Hochberg FDR < 0.05); "—" marks rules specified by expert elicitation and calibrated on the training split only (Section 2.5). Thresholds on continuous cues were set at the value maximising Youden's J on the training split; persistence windows (7 days for temperature anomaly) were chosen by scanning 3–14-day windows and taking the shortest window at which the AUC plateaued. [M] = measured on the mosquito corpus; [I] = illustrative template, not evaluated on measured data.

2. Supplementary file inventory

File / folder	What it contains	Format	Manuscript item
File_S1_Wing_Images_and_Landmarks	Raw grayscale wing micrographs of the primary corpus (n = 1,200; Aedes albopictus	JPG images + CSV / JSON tables	Section 2.1–2.2, Table 5, Figures

	and Anopheles spp., 2020–2024) together with the digitized landmark and semilandmark coordinates used for model training and for the accuracy assessment reported in Table 5.		5–6
File_S2_Alert_JSON	Alert records in JSON format emitted by warn_engine.py, including the worked example from raw image to issued alert shown in Figure 6.	JSON (WingShape-Warn/alert/v1)	Section 2.5–2.6, Figure 6, Section 3.7
File_S3_UNet_Checkpoint	Trained weights and configuration file of the U-Net landmark detector used in Step 2.	PyTorch .pt + JSON + .py	Section 2.2, Table 5
README.txt	File inventory, directory structure, software versions and the commands required to reproduce the pipeline.	Plain text	Section 2.8, Data Availability Statement
File_S4_Readable_Documentation (this document)	Directly readable rendering of Table S1 and of File_S1–File_S3, plus the README content.	DOCX	Response to Reviewer 2

3. File_S1_Wing_Images_and_Landmarks

"Raw grayscale wing micrographs of the primary corpus (n = 1,200; Aedes albopictus and Anopheles spp., 2020–2024) together with the digitized landmark and semilandmark coordinates used for model training and for the accuracy assessment reported in Table 5."

3.1 Contents

```
File_S1_Wing_Images_and_Landmarks/
├── images/                          1,200 grayscale wing micrographs, .jpg, 1200 px long edge
│                                    naming: WSW_<zone>_<taxon>_<sex>_<nnn>.jpg
├── landmark_coordinates.csv         1,200 rows × 25 cols  (specimen_id + 12 landmarks × x,y)
├── semilandmark_coordinates.csv     1,200 rows × 73 cols  (specimen_id + 36 semilandmarks × x,y)
├── landmark_configuration.json      12-landmark scheme + 3 semilandmark curves + units
└── specimen_metadata.csv            1,200 rows × 16 cols  (zone, taxon, sex, date, grid, PCs, CS)
```

3.2 Landmark configuration (12 landmarks)

Landmark(s)	Anatomical definition
1–2	Humeral plate / alula junction (wing base, left and right)
3	Costal break (C, proximal)
4–5	Crossvein h / radial sector fork
6	Radial–medial crossvein (r-m)
7–8	Medial–cubital crossvein (m-cu) and medial fork
9–10	Cubital fork (CuA1 / CuA2)
11	Medial–cubital (m-cu) distal insertion
12	Wing apex (R4+5 terminus)

Coordinate units: pixels, origin at the top-left of the image. Scale: 0.5 mm scale bar = 0.22 × image width (see the px_to_mm column of specimen_metadata.csv; 0.004058 mm per pixel at 4.8 px μm^{-1}). Digitised by the U-Net (ResNet-34 encoder), 5-fold cross-validation grouped by collection site; mean radial error 1.34 ± 0.41 px against manual digitisation.

3.3 Sliding semilandmark curves (36 points)

Curve	Between landmarks	Number of points	Sliding criterion
1	3 → 4	12	Minimise Procrustes distance (geomorph "digitsurface")
2	5 → 6	12	Minimise Procrustes distance
3	9 → 11	12	Minimise Procrustes distance

3.4 Coordinate-file layout

Column	Type	Description
specimen_id	string	WSW_<climate zone>_<taxon>_<sex>_<number>, e.g. WSW_C1_AED_F_001
lm1_x … lm12_y	float	Pixel coordinates of the 12 landmarks, origin top-left
（semilandmark file）<br>slm1_x … slm36_y	float	Pixel coordinates of the 36 sliding semilandmarks, ordered by curve

3.5 Specimen metadata fields

Column	Description
specimen_id	Join key, identical to the coordinate files
climate_zone / zone_label	C1–C5 along the temperature gradient (e.g. C1 = Cool temperate)
isotherm_C	Mean annual isotherm of the zone (20.0–28.0 °C)
taxon / sex	Aedes_albopictus or Anopheles spp.; F / M
collection_date	ISO date of capture, 2020–2024
grid_cell	Surveillance grid cell (city + cell number, e.g. Zhanjiang-07)
temp_anomaly_C	Temperature anomaly of the collection week relative to the 1991–2020 mean
centroid_size_mm	Centroid size in millimetres (separate size variable, see Section 2.2)
PC1 / PC2 / PC3	Procrustes shape scores in SD units relative to the training consensus
image_file / px_to_mm	Relative image path and the millimetres-per-pixel conversion factor

3.6 Reading the files without the pipeline (Python)

```
import pandas as pd

lm   = pd.read_csv("File_S1_Wing_Images_and_Landmarks/landmark_coordinates.csv")
meta = pd.read_csv("File_S1_Wing_Images_and_Landmarks/specimen_metadata.csv")

# 12 landmarks -> (n_specimens, 12, 2) array
coords = lm.filter(regex=r"^lm\d+_[xy]$").to_numpy().reshape(len(lm), 12, 2)

# pixels -> millimetres, using the per-image scale factor
scale     = meta.set_index("specimen_id").loc[lm.specimen_id, "px_to_mm"].to_numpy()
coords_mm = coords * scale[:, None, None]

print(lm.specimen_id.iloc[0], coords_mm.shape)   # WSW_C1_AED_F_001 (12, 2)
```

3.7 Reading the files without the pipeline (R / geomorph)

```
library(geomorph)

lm   <- read.csv("File_S1_Wing_Images_and_Landmarks/landmark_coordinates.csv")
meta <- read.csv("File_S1_Wing_Images_and_Landmarks/specimen_metadata.csv")

# specimen x (x,y) matrix -> geomorph array (p landmarks, k dimensions, n specimens)
A <- aperm(array(as.matrix(lm[, -1]), dim = c(nrow(lm), 12, 2)), perm = c(2, 3, 1))

# semilandmark curves: column indices of the 36 sliding points, 12 per curve
slm <- read.csv("File_S1_Wing_Images_and_Landmarks/semilandmark_coordinates.csv")
curves <- list(1:12, 13:24, 25:36)

gpa  <- gpagen(A, curves = curves, ProcD = TRUE)
pca  <- gm.prcomp(gpa$coords)
plot(gpa)          # aligned specimens
plot(pca, axis1 = 1, axis2 = 2)   # PC1 (42.3%) vs PC2 (18.7%), cf. Table 2a
```

4. File_S2_Alert_JSON

"Alert records in JSON format emitted by warn_engine.py, including the worked example from raw image to issued alert shown in Figure 6."

4.1 Contents

```
File_S2_Alert_JSON/
├── alert_schema.json                     WingShape-Warn/alert/v1 field specification
├── alert_WSW-ALERT-2024-0731-ZJ07.json   worked example of Figure 6, verbatim engine output
└── alerts_2023-2024/                     37 prospectively recorded alerts (lead-time sample)
```

4.2 Alert schema, field by field

Field	Type / content
alert_id	String, format WSW-ALERT-YYYY-MMDD-<grid>
generated_at	ISO-8601 timestamp with UTC offset
pipeline_version	String (1.0.0 in this release)
specimen	Object: specimen_id, taxon, sex, grid_cell, climate_zone, collection_date
input	Object: image_file, scale_px_to_mm
step2_landmarks	Object: model, checkpoint, n_landmarks, n_semilandmarks, mean_confidence, routed_to_manual_review
step3_shape_scores	Object: PC1–PC3, centroid_size_mm, centroid_size_pct_vs_C1_mean, mahalanobis_D2, reference
step4_evidence_chain	Array of {from, rel, to, probability}
step5_rule_evaluation	Object: rule_id, conditions[], conditions_met, conditions_total, support, confidence, lift
step6_decision	Object: alert_level, probability_outbreak_8wk, lead_time_weeks, recommended_actions[], operating_point
validation	Object: ground_truth_recorded, trap_count_exceeded_seasonal_threshold, weeks_to_threshold_exceedance, note

4.3 The worked example of Figure 6, rendered as a table

Block	Field	Value
specimen	specimen_id / taxon / sex	WSW_C5_AED_F_042 / Aedes albopictus / F
specimen	grid_cell / climate_zone / date	Zhanjiang-07 / C5 / 2024-07-31
input	image_file / scale	images/WSW_C5_AED_F_042.jpg / 0.004058 mm px^{-1}
step2	model / checkpoint	unet_resnet34_landmark / unet_resnet34_landmark.pt
step2	landmarks + semilandmarks	12 + 36
step2	mean_confidence / manual review	0.971 / false
step3	PC1 / PC2 / PC3	+2.41 SD / −1.12 SD / +0.58 SD
step3	centroid size / vs C1 mean	3.13 mm / −8.7%
step3	Mahalanobis D^2 (reference n = 1,200)	6.84
step4	evidence chain	WingPC1_High —PREDICTS→ Abundance_Up (0.88); TempAnom_+2C —TRIGGERED_BY→ WingPC1_High

		(0.91); UrbanLand_High —TRIGGERED_BY→ WingPC1_High (0.76); Abundance_Up —PREDICTS→ DengueOutbreak (0.86)
step5	rule_id / conditions met	R03_dengue_warmzone / 3 of 5
step5	conditions	WingPC1 shift > 2 SD (2.41 vs 2.0) ✓; centroid-size reduction > 8% (−8.7 vs −8.0) ✓; temperature anomaly > +2 °C for 7 d (2.4 vs 2.0) ✓; virus detected in pool (null) ✗; rainfall anomaly > +1 SD (0.4 vs 1.0) ✗
step5	support / confidence / lift	0.34 / 0.78 / 1.9
step6	alert_level / P(outbreak | 8 wk)	YELLOW / 0.62
step6	lead time / operating point	5 weeks (range 4–6) / Youden J 0.75, sensitivity 0.84, specificity 0.91
step6	recommended actions	Add one ovitrap to grid cell Zhanjiang-07; larval survey of containers within 200 m; re-sample wing morphology in 14 days
validation	ground truth	Trap counts in the same grid cell exceeded the seasonal threshold 5 weeks after the alert

4.4 The emitted JSON (verbatim, condensed for printing)


```
{
  "schema": "WingShape-Warn/alert/v1",
  "alert_id": "WSW-ALERT-2024-0731-ZJ07",
  "generated_at": "2024-07-31T09:14:22+08:00",
  "pipeline_version": "1.0.0",
  "specimen": { "specimen_id": "WSW_C5_AED_F_042", "taxon": "Aedes albopictus",
                "sex": "F", "grid_cell": "Zhanjiang-07",
                "climate_zone": "C5", "collection_date": "2024-07-31" },
  "step3_shape_scores": { "PC1": 2.41, "PC2": -1.12, "PC3": 0.58,
                          "centroid_size_mm": 3.13,
                          "centroid_size_pct_vs_C1_mean": -8.7,
                          "mahalanobis_D2": 6.84,
                          "reference": "training consensus, n = 1,200" },
  "step5_rule_evaluation": { "rule_id": "R03_dengue_warmzone",
                             "conditions_met": 3, "conditions_total": 5,
                             "support": 0.34, "confidence": 0.78, "lift": 1.9 },
  "step6_decision": { "alert_level": "YELLOW", "probability_outbreak_8wk": 0.62,
                      "lead_time_weeks": { "point": 5.0, "range": [4, 6] } },
  "validation": { "ground_truth_recorded": true,
                  "trap_count_exceeded_seasonal_threshold": true,
                  "weeks_to_threshold_exceedance": 5 }
}
```


4.5 Regenerating an alert

```
# one specimen, end to end (Step 2 -> Step 6)
python scripts/preprocess.py  --input images/WSW_C5_AED_F_042.jpg --out work/wing.png
python scripts/landmark_cnn.py --checkpoint File_S3_UNet_Checkpoint/unet_resnet34_landmark.pt \
                               --input work/wing.png --out work/wing_landmarks.csv
python scripts/warn_engine.py  --landmarks work/wing_landmarks.csv \
                               --rules rules/rule_base.json \
                               --graph export/neo4j_subgraph.csv \
                               --out File_S2_Alert_JSON/alert_WSW-ALERT-2024-0731-ZJ07.json
```

```
# validates any alert record against the schema
python scripts/validate_alert.py --schema File_S2_Alert_JSON/alert_schema.json \
                                 --alert  File_S2_Alert_JSON/alert_WSW-ALERT-2024-0731-ZJ07.json
```

5. File_S3_UNet_Checkpoint

"Trained weights and configuration file of the U-Net landmark detector used in Step 2."

5.1 Contents

```
File_S3_UNet_Checkpoint/
├── unet_resnet34_landmark.pt    trained weights (48 output heatmap channels: 12 + 36)
├── training_config.json         architecture, hyper-parameters and measured accuracy
└── model_architecture.py        the exact nn.Module definition used for the checkpoint
```

5.2 Architecture

```
class UNetLandmark(nn.Module):
    """Encoder : ResNet-34 (ImageNet weights), 1-channel input
       Decoder : U-Net upsampling with skip connections
       Head    : 1x1 conv -> 12 + 36 heatmaps -> differentiable soft-argmax"""
    def __init__(self, n_landmarks=12, n_semilandmarks=36, pretrained=True):
        super().__init__()
        enc = models.resnet34(weights="IMAGENET1K_V1" if pretrained else None)
        enc.conv1 = nn.Conv2d(1, 64, 7, stride=2, padding=3, bias=False)   # grayscale
        self.enc1 = nn.Sequential(enc.conv1, enc.bn1, enc.relu)
        self.enc2 = nn.Sequential(enc.maxpool, enc.layer1)   # 1/4
        self.enc3, self.enc4, self.enc5 = enc.layer2, enc.layer3, enc.layer4
        self.up4, self.up3 = self._up(512, 256), self._up(256, 128)
        self.up2, self.up1 = self._up(128, 64),  self._up(64, 64)
        self.head = nn.Conv2d(64, n_landmarks + n_semilandmarks, 1)

    def forward(self, x):
        e1 = self.enc1(x); e2 = self.enc2(e1)
        e3, e4, e5 = self.enc3(e2), self.enc4(e3), self.enc5(e4)
        d = self.up4(e5) + e4; d = self.up3(d) + e3
        d = self.up2(d)  + e2; d = self.up1(d)  + e1
        return self.head(d)
```

5.3 Training configuration

Item	Value
Model	U-Net, ResNet-34 encoder (ImageNet pre-trained), heatmap regression
Task	12 landmarks + 36 sliding semilandmarks
Input	Grayscale, resized to 512 × 512 px, CLAHE + per-image z-score normalisation
Output	Gaussian heatmaps, $\sigma = 2$ px; coordinates recovered by differentiable soft-argmax
Optimiser / schedule	AdamW, lr 1×10^{-3}, cosine decay, weight decay 1×10^{-4}
Batch / epochs / early stopping	16 / ≤ 200 / patience 30 on the validation fold
Loss	MSE on the heatmaps + 0.1 × soft-argmax coordinate term
Augmentation	Rotation ±15°; horizontal and vertical flips; scale 0.9–1.1; brightness ±10%; Gaussian noise $\sigma = 0.01$; elastic deformation $\alpha = 20$
Cross-validation	5-fold, grouped by collection site (no site in both train and validation)
Hardware / runtime	1 × NVIDIA RTX 3090 (24 GB); training ≈ 3.4–6.4 h; inference 0.9 s per wing
Weights file	unet_resnet34_landmark.pt

5.4 Measured accuracy (Table 5)

Metric	Value
Mean radial error vs manual digitisation	1.34 ± 0.41 px (pre-registered tolerance 2 px)
95th percentile radial error	3.1 px
Success detection rate	87.6% below 2 px; 98.2% below 4 px
Agreement in shape space	Procrustes distance 0.0112; PC1 correlation r = 0.994; ICC(2,1) = 0.987
Human inter-operator repeatability	1.12 ± 0.33 px (second operator, 60 wings)
Downstream impact	ΔAUC (network − manual landmarks) = −0.005 on the abundance model
Manual-review rate	2.3% of wings flagged (Mahalanobis $D^2 > 3$ on residuals)

Radial error is given in pixels at a scanning resolution of 4.8 px μm^{-1} (scale bar 0.5 mm). The detector error is only marginally larger than the inter-operator repeatability of expert human digitisation.

5.5 Loading the checkpoint

```
import torch
from model_architecture import UNetLandmark

model = UNetLandmark(n_landmarks=12, n_semilandmarks=36, pretrained=False)
state = torch.load("File_S3_UNet_Checkpoint/unet_resnet34_landmark.pt",
                   map_location="cpu")
model.load_state_dict(state)
model.eval()

# x: (1, 1, 512, 512) float tensor, CLAHE + z-scored
with torch.no_grad():
    heatmaps = model(x)            # (1, 48, 512, 512)

# soft-argmax -> (48, 2) coordinates in pixels
h, w    = heatmaps.shape[-2:]
weights = torch.softmax(heatmaps.flatten(-2), dim=-1).view(48, h * w)
grid    = torch.stack(torch.meshgrid(torch.arange(w), torch.arange(h),
                                     indexing="xy"), dim=-1).float().view(h * w, 2)
coords  = weights @ grid          # 12 landmarks then 36 semilandmarks
```

6. README.txt (reproduced)

"File inventory, directory structure, software versions and the commands required to reproduce the pipeline."

6.1 File inventory

File	Records / items	Purpose
File_S1_Wing_Images_and_Landmarks/images/	1,200 JPG	Model input; the measured corpus
File_S1_Wing_Images_and_Landmarks/landmark_coordinates.csv	1,200 rows	Network-derived landmark coordinates used in GPA / PCA
File_S1_Wing_Images_and_Landmarks/semilandmark_coordinates.csv	1,200 rows	36 sliding points on three vein curves
File_S1_Wing_Images_and_Landmarks/landmark_configuration.json	1 file	Landmark definitions, curves, units and scale
File_S1_Wing_Images_and_Landmarks/specimen_metadata.csv	1,200 rows	Stratification, environment, PCs, centroid size
File_S2_Alert_JSON/alert_schema.json	1 file	Alert record specification v1
File_S2_Alert_JSON/alert_WSW-ALERT-2024-0731-ZJ07.json	1 record	Worked example of Figure 6
File_S2_Alert_JSON/alerts_2023-2024/	37 records	Prospective lead-time sample
File_S3_UNet_Checkpoint/unet_resnet34_landmark.pt	1 checkpoint	Step 2 inference
File_S3_UNet_Checkpoint/training_config.json	1 file	Hyper-parameters and measured accuracy
File_S3_UNet_Checkpoint/model_architecture.py	1 file	Model definition
File_S4_WingShape-Warn_Readable_Documentation.docx	1 document	Directly readable rendering of everything above

6.2 Directory structure

```
WingShape-Warn_supplementary/
├── README.txt
├── File_S4_WingShape-Warn_Readable_Documentation.docx
├── File_S1_Wing_Images_and_Landmarks/
│   ├── images/
│   ├── landmark_coordinates.csv
│   ├── semilandmark_coordinates.csv
│   ├── landmark_configuration.json
│   └── specimen_metadata.csv
├── File_S2_Alert_JSON/
│   ├── alert_schema.json
│   ├── alert_WSW-ALERT-2024-0731-ZJ07.json
│   └── alerts_2023-2024/
└── File_S3_UNet_Checkpoint/
    ├── unet_resnet34_landmark.pt
    ├── training_config.json
    └── model_architecture.py
```

6.3 Software versions

Component	Version used	How to verify
Python	3.10.x (≥3.10 required)	python --version

PyTorch	2.x (CUDA build recommended)	python -c "import torch; print(torch.__version__)"
torchvision	matching the PyTorch build	python -c "import torchvision; print(torchvision.__version__)"
NumPy / pandas / scikit-learn / matplotlib	1.26.x / 2.x / 1.4.x / 3.8.x	python -c "import numpy, pandas, sklearn, matplotlib; print(numpy.__version__, pandas.__version__, sklearn.__version__, matplotlib.__version__)"
R	4.4.x (≥4.4 required)	R --version
geomorph	4.0.x	Rscript -e 'packageVersion("geomorph")'
ggplot2	3.5.x	Rscript -e 'packageVersion("ggplot2")'
MorphoJ (stand-alone, GUI)	1.07a	Help → About
tpsDig2 (stand-alone, GUI)	2.32	Help → About
Neo4j	5.x Community	neo4j --version
OS	Ubuntu 22.04 LTS; Windows 11	—
GPU	NVIDIA RTX 3090 24 GB, CUDA 11.8	nvidia-smi

Versions are given as the minor line actually used by the authors; the pipeline requires only the minimum versions stated in Section 2.8 (Python 3.10+, R 4.4+). Run the commands in the last column to print the exact versions of your own environment and paste them into your copy of README.txt.

6.4 Commands required to reproduce the pipeline

```
# 0 - environment -------------------------------------------------------
python -m venv .venv && source .venv/bin/activate
pip install -r requirements.txt            # torch, torchvision, numpy, pandas, scikit-learn,
matplotlib
Rscript -e 'install.packages(c("geomorph","ggplot2"))'

# 1 - Step 2: preprocessing and landmark detection ----------------------
python scripts/preprocess.py   --input File_S1_Wing_Images_and_Landmarks/images \
                               --out   work/preprocessed
python scripts/landmark_cnn.py --checkpoint File_S3_UNet_Checkpoint/unet_resnet34_landmark.pt \
                               --input work/preprocessed --out work/landmarks.csv

# 2 - Step 3: GPA, PCA, PLS, allometry ----------------------------------
Rscript scripts/gm_analysis.R  --landmarks work/landmarks.csv \
                               --metadata File_S1_Wing_Images_and_Landmarks/specimen_metadata.csv \
                               --out work/shape_scores.csv
python scripts/apriori_miner.py --shape work/shape_scores.csv --min-support 0.3 \
                                --min-confidence 0.7 --out work/rules.csv

# 3 - Step 4: knowledge graph -------------------------------------------
python scripts/kg_builder.py   --rules work/rules.csv --out work/kg_export/
neo4j-admin database import full --nodes=work/kg_export/nodes.csv \
                                  --relationships=work/kg_export/edges.csv

# 4 - Step 5 / 6: rule base, alert emission, report ---------------------
python scripts/warn_engine.py  --shape work/shape_scores.csv --rules rules/rule_base.json \
                               --out File_S2_Alert_JSON/
```

```
Rscript -e 'rmarkdown::render("scripts/report_template.Rmd")'

# 5 - Step 7: retrospective forecast and ROC / ablation -----------------
python scripts/validate.py     --shape work/shape_scores.csv \
                               --metadata File_S1_Wing_Images_and_Landmarks/specimen_metadata.csv \
                               --models E S E+S E+S_perm --bootstrap 1000

# 6 - reproduce every figure -------------------------------------------
Rscript scripts/make_figures.R            # geometric-morphometric panels (R / ggplot2)
python scripts/make_figures.py            # ROC, decision tree, boxplots, alert cards (Python)
```

7. Where each file is used in the manuscript

Manuscript item	Source file
Table 5 — landmark-detector configuration and measured accuracy	File_S3_UNet_Checkpoint/training_config.json; File_S1 landmark coordinates vs manual digitisation
Table 2a — PCA eigenvalues (PC1 42.3%, PC2 18.7%, PC3 12.4%)	File_S1 specimen_metadata.csv + landmark_coordinates.csv (GPA in gm_analysis.R)
Table 6 — ablation (E, S, E+S, E+S_perm)	File_S1 specimen_metadata.csv, scripts/validate.py
Figure 5 — reference versus warm-zone wing, deformation grid, zone boxplots	File_S1 images/WSW_C1_AED_F_001.jpg and images/WSW_C5_AED_F_042.jpg, scripts/make_figures.R / .py
Figure 6 — worked example, image to alert	File_S1 images/WSW_C5_AED_F_042.jpg; File_S2 alert_WSW-ALERT-2024-0731-ZJ07.json
Section 3.7 — lead time 4.2 ± 1.6 weeks	File_S2 alerts_2023-2024/ (37 prospectively recorded alerts)
Table S1 — 18-rule early-warning rule base	Section 1 of this document; rules/rule_base.json consumed by warn_engine.py
